# The androgen receptor—lncRNA*SAT1-*AKT-p15 axis mediates androgen-induced cellular senescence in prostate cancer cells

**DOI:** 10.1038/s41388-021-02060-5

**Published:** 2021-10-19

**Authors:** Kimia Mirzakhani, Julia Kallenbach, Seyed Mohammad Mahdi Rasa, Federico Ribaudo, Martin Ungelenk, Marzieh Ehsani, Wenrong Gong, Nikolaus Gassler, Mirjam Leeder, Marc-Oliver Grimm, Francesco Neri, Aria Baniahmad

**Affiliations:** 1 Institute of Human Genetics, Jena University Hospital, Jena, Germany; 2 Leibniz Institute on Aging, Jena, Germany; 3 Medical College, Hubei University of Arts and Science, Xiangyang, China; 4 Section of Pathology, Institute of Forensic Medicine, Jena University Hospital, Jena, Germany; 5 Department of Adult and Pediatric Urology, Jena University Hospital, Jena, Germany; 6 Present Address: SCW Medicath LTD, Baolong industrial Town, Shenzhen, China

**Keywords:** Cancer, Prostate cancer

## Abstract

The bipolar androgen therapy (BAT) to treat prostate cancer (PCa) includes cycles of supraphysiological androgen levels (SAL) under androgen-deprivation therapy (ADT). We showed previously that SAL induces cellular senescence in androgen-sensitive PCa cells and in ex vivo-treated patient PCa tumor samples. Here, we analyzed the underlying molecular pathway and reveal that SAL induces cellular senescence in both, castration-sensitive (CSPC) LNCaP and castration-resistant PCa (CRPC) C4-2 cells through the cell cycle inhibitor p15^INK4b^ and increased phosphorylation of AKT. Treatment with the AKT inhibitor (AKTi) potently inhibited SAL-induced expression of p15^INK4b^ and cellular senescence in both cell lines. Proximity-ligation assays (PLA) combined with high-resolution laser-scanning microscopy indicate that SAL promotes interaction of endogenous androgen receptor (AR) with AKT in the cytoplasm as well as in the nucleus detectable after three days. Transcriptome sequencing (RNA-seq) comparing the SAL-induced transcriptomes of LNCaP with C4-2 cells as well as with AKTi-treated cell transcriptomes revealed landscapes for cell senescence. Interestingly, one of the identified genes is the lncRNA*SAT1*. SAL treatment of native patient tumor samples ex vivo upregulates lncRNA*SAT1*. In PCa tumor tissues, lncRNA*SAT1* is downregulated compared with nontumor tissues of the same patients. Knockdown indicates that the lncRNA*SAT1* is crucial for SAL-induced cancer-cell senescence as an upstream factor for pAKT and for p15^INK4b^. Further, knockdown of lncRNA*SAT1* enhances cell proliferation by SAL, suggesting that lncRNA*SAT1* serves as a tumor suppressor at SAL. Interestingly, immunoprecipitation of AR detected lncRNA*SAT1* as an AR-interacting partner that regulates AR target-gene expression. Similarly, RNA-ChIP experiments revealed the interaction of AR with lncRNA*SAT1* on chromatin. Thus, we identified a novel AR-lncRNA*SAT1*-AKT-p15^INK4b^ signaling axis to mediate SAL-induced cellular senescence.

## Introduction

The normal prostate and prostate cancer (PCa) are androgen-regulated tissues in which physiological and intratumoral levels of androgens promote cell proliferation. The cancerous prostate is a major life-threatening cancer in men [1] ranking as the second leading cause of cancer death in the United States. PCa develops from an androgen-dependent, castration-sensitive (CSPC) to a castration-resistant (CRPC) tumor that grows despite-androgen deprivation therapy (ADT). The androgen receptor (AR) regulates the growth of CSPC and CRPC, therefore being an important drug target. Inhibition of AR-mediated transactivation by ADT and antiandrogens results in tumor-growth inhibition until therapy resistance occurs. Interestingly, PCa exhibits a biphasic growth response toward androgen concentrations. Low, physiological androgen levels (LAL) promote growth, whereas, paradoxically, high, supraphysiological androgen levels (SAL) inhibit also PCa cell proliferation in preclinical models [2–5].

In line with that, enhancing testosterone levels to a supraphysiological level results in reduced disease progression [6]. Accordingly, low dose of androgens in individuals is associated with increased PCa risk, which suggests a tumor-suppressive role by higher androgen levels [7, 8]. This is further supported by ongoing clinical trials that use intermittent pharmacologic SAL that might be beneficial for a subset of PCa patients [9–11]. In clinical trials of bipolar androgen therapy (BAT), metastatic CRPC patients continuously are maintained on ADT via luteinizing hormone-releasing hormone-agonist therapy and given intermittent cycles of pharmacologic SAL via intramuscular injection. This approach is based on a serial bipolar cycling between pharmacological SAL followed by a rapid decline to castrate levels of androgens and is coupled to the facts that during prostatic carcinogenesis the AR acquires oncogenic activity [9–14].

Thus, the level/concentration of androgens seems to be very important and might dictate the outcome of cellular responses of PCa cells. Accordingly, preclinical mouse models suggest that the AR exhibits both, proliferation-promoting and tumor-suppressive functions [15, 16]. Interestingly along with growth inhibition by SAL, SAL induces cellular senescence in CSPC cancer cells. However, the underlying molecular mechanism of SAL-mediated growth inhibition of PCa is less examined and not well understood.

Induction of cell senescence by SAL is AR-dependent. AR negative human PCa cells does not respond to SAL, whereas an inducible AR-expression system in AR-negative cells renders cells sensitive to SAL-mediated cell senescence [4, 17].

The AR is a member of steroid and nuclear hormone-receptor superfamily. Similar to other members of this family, the AR is a ligand-controlled transcription factor. In the absence of ligand, the AR is bound by heat-shock proteins and is localized in the cytoplasm. Upon androgen binding (agonist DHT or R1881), the AR becomes activated and undergoes a conformational change. The heat-shock proteins dissociate from the receptor that unmasks the nuclear-localization signal of the AR, which induces the AR translocation into the nucleus. Classically, the activated AR subsequently associates with chromatin and binds directly to androgen-response elements, known as genomic signaling. This leads to modulation of expression of neighboring genes, e.g., direct positively regulated AR genes such as *KLK3*, encoding PSA; the diagnostic marker prostate-specific antigen; and *FKBP5* or directly repressed genes such as the telomerase subunit hTERT [18].

However, in addition to this genomic action of AR, androgens mediate a rapid nongenomic signaling by phosphorylation of signal-transduction factors such as the non-tyrosine kinase Src, the prosurvival factor AKT/PKB, and the Ras/Raf signal transducer MAPK within few minutes [19]. Notably the androgen-induced phosphorylation disappears within one hour [19]. Interestingly, the AKT inhibitor, AKTi, inhibits SAL-mediated cell senescence in LNCaP cells, whereas inhibition of the MAPK pathway did not interfere with the SAL-mediated cell senescence [4], suggesting that the AR-AKT signaling mediates androgen-induced cell senescence.

It is still unclear whether the AR-AKT interaction is also detectable after a long-term treatment. Moreover, the intracellular localization of the AR-AKT interaction after a long-term treatment has not yet been identified.

Here, we analyzed a molecular pathway for androgen-induced cellular senescence involving AKT. We identified an interaction of endogenously expressed AR-AKT being detectable after three days of SAL treatment using PLA assays. RNA-seq was used to identify the SAL-induced and AKT-reversal senescence transcriptome signature of both human CSPC LNCaP and CRPC C4-2 cells. The overlap of transcriptome landscapes of both cell lines resulted in 33 genes being commonly upregulated by SAL and downregulated by AKTi. Thereby, the lncRNA*SAT1* was functionally analyzed for its role in SAL-induced cellular senescence. Based on knockdown and functional experiments, we propose that the lncRNA*SAT1* is upstream of the AR-AKT interaction and induction of p15^INK4b^ to mediate SAL-induced cellular senescence. Interestingly, the lncRNA*SAT1* interacts with AR on chromatin and is part of the AR signaling.

## Results

### p15^INK4b^ mediates cellular senescence induced by SAL

**Fig. 1:**
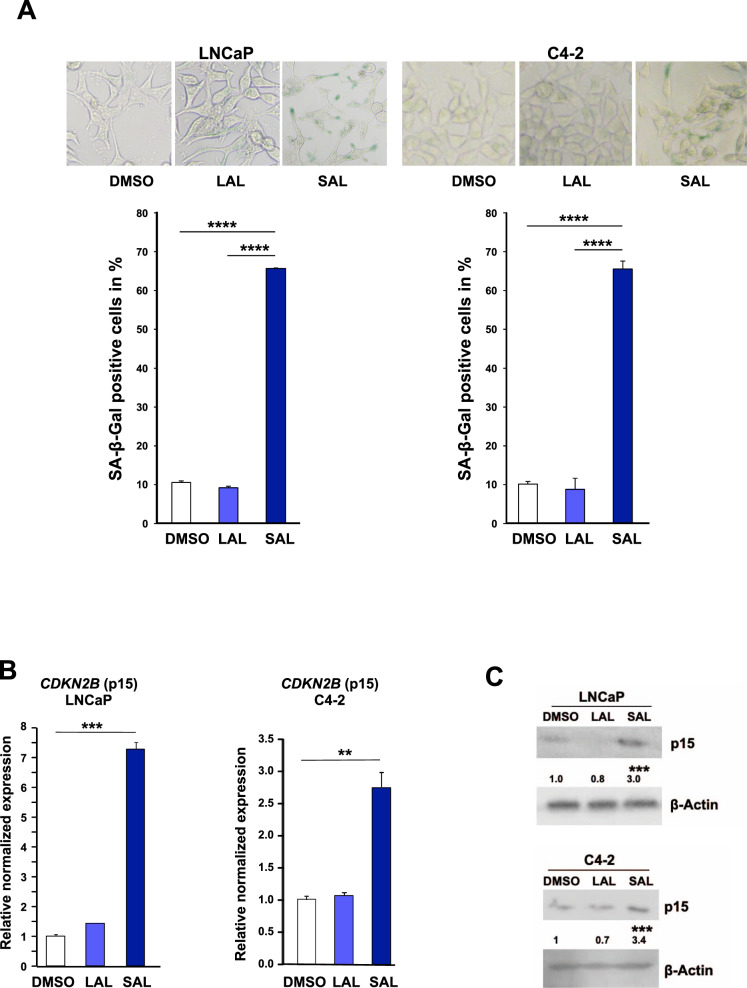
SAL induces cellular senescence in both CSPC and CRPC cells associated with an increase of *CDKN2B* / p15^INK4b^ levels. Androgen-dependent LNCaP and castration-resistant C4-2 PCa cell lines were incubated for 72 h with DMSO, as solvent control, 1 pM R1881 (methyltrionolone) defined as low androgen levels (LAL), or 1 nM R1881 as supraphysiological androgen level (SAL) [4]. **A** Upper panel: Representative pictures of SA-β-Gal staining of LNCaP and C4-2 cells at 200x magnification. Lower panel: quantification of SA-β-Gal-positive stained cells. Bar graphs represent mean ± SD (*n* = 3). **B** Detection of *CDKN2B* mRNA expression by qRT-PCR normalized to the house-keeping genes *TBP* and *GAPDH*. Bar graphs represent mean ± SEM (*n* = 3). **C** Changes of p15^INK4B^ protein level were detected by Western blotting. Values of control samples were set arbitrarily as 1. Quantification of bands was performed by LabImage D1 normalized to the loading control β-actin (*n* = 3). **p ≤ 0.01, ***p ≤ 0.001, ****p ≤ 0.0001.

LNCaP and C4-2 cells as model system for CSPC and CRPC, respectively, were treated either with 1pM (LAL) or 1 nM (SAL) methyltrienolone (R1881) for three days. R1881 is less metabolized than dihydrotestosterone (DHT). Further, DHT metabolites are known to activate other nuclear receptors such as estrogen-receptor beta [20]. Hence, R1881 exhibits a better AR specificity and thus causes less unspecific side effects. Induction of cell senescence was analyzed by detection of the senescence-associated β-galactosidase (SA-β-Gal) activity and expression of *CDKN2B* encoding the cell cycle inhibitor p15^INK4b^. The data indicate that SAL induces cellular senescence in both LNCaP and C4-2 cell lines (Fig. 1A). In line with this, the expression of the CDK inhibitor *CDKN2B*/p15^INK4b^ was induced specifically by SAL treatment in both cell lines at mRNA and protein level (Fig. 1B, C).

**Fig. 2:**
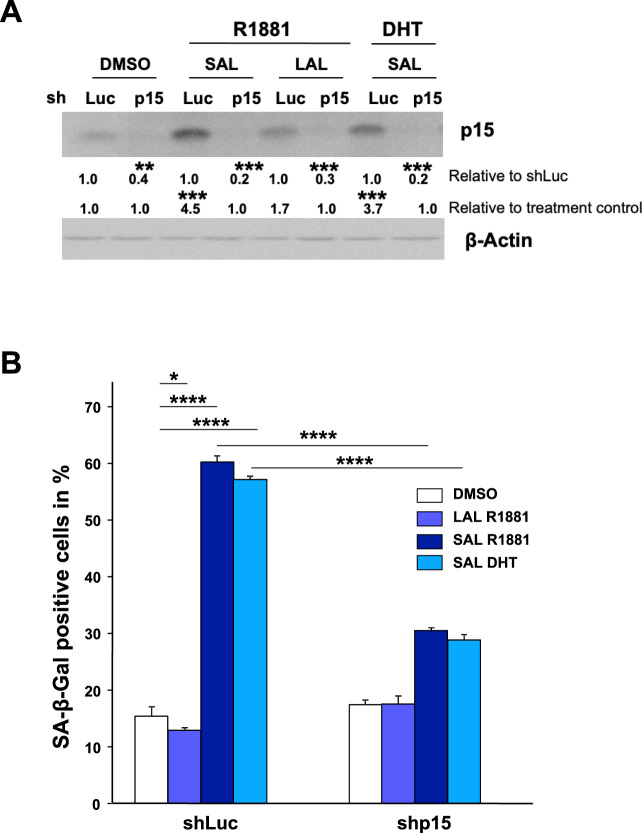
Knockdown of *CDKN2B*/p15^INK4B^ inhibits SAL-induced cellular senescence. **A** *CDKN2B*/p15^INK4B^ knockdown was confirmed by Western blotting. Quantification of bands was performed by LabImage D1 normalized to the loading control β-Actin. Data are presented as means ± SD (*n* = 3). **B** Quantification of SA-β-Gal positive stained LNCaP cells with and without *CDKN2B* knockdown. Numbers indicate the mean ± SD (*n* = 3). *p ≤ 0.05, ****p ≤ 0.0001.

To analyze whether the increased *CDKN2B*/p15^INK4b^ levels are not only associated but regulate the AR-mediated senescence pathway, we performed knockdown experiments. The p15^INK4b^ knockdown was verified by Western blotting (Fig. 2A).

The SAL-mediated cellular senescence levels induced by either R1881 or DHT are strongly reduced in the *CDKN2B-*knockdown cells (Fig. 2B). This suggests that *CDKN2B*/p15^INK4b^ is a downstream factor of SAL-induced cell-senescence pathway. Moreover, it indicates that p15^INK4b^ is a key factor of the AR axis to induce cellular senescence by SAL.

### SAL induces interaction of AR with AKT

**Fig. 3:**
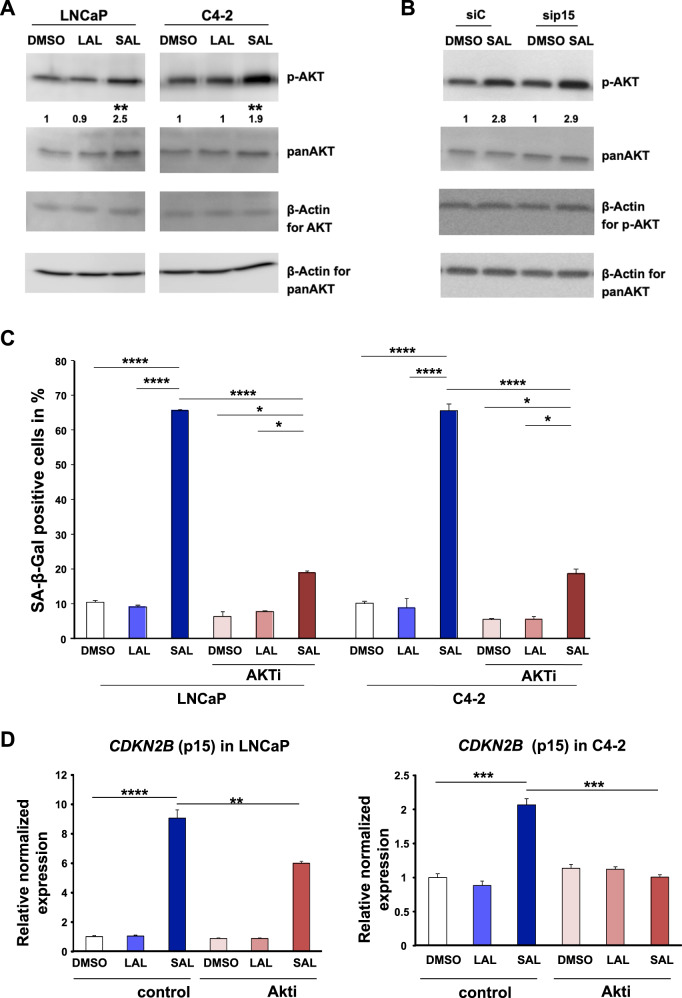

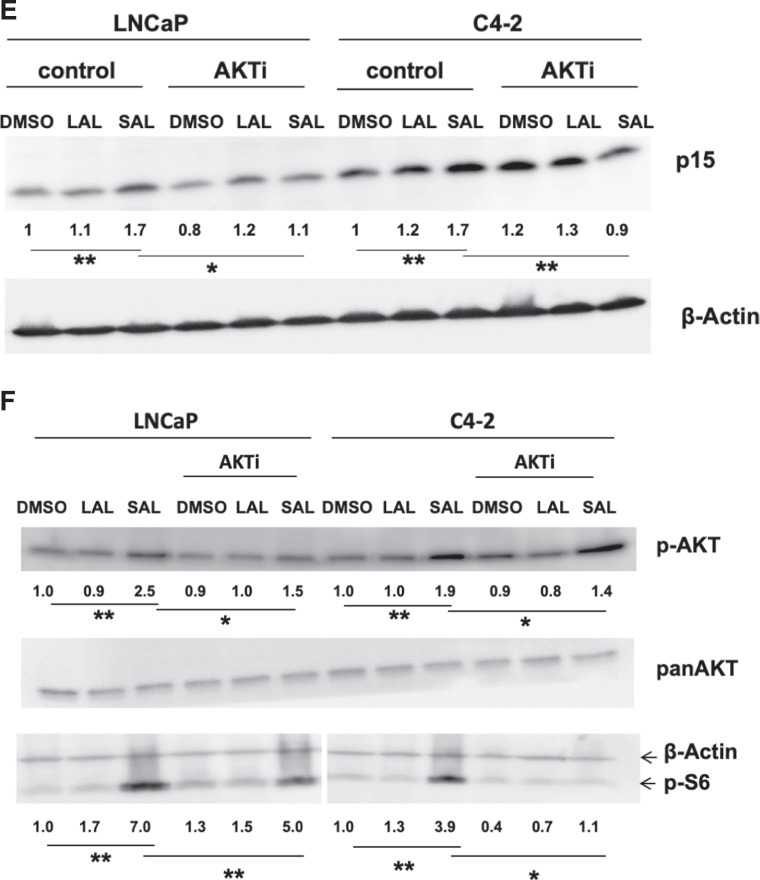
The AKT inhibitor (AKTi) reduces the androgen-induced cellular senescence in both CSPC and CRPC cells. LNCaP cells were treated for 72 h with SAL, LAL, or 0.1% DMSO as solvent control. **A** The level of p-AKT is enhanced in both LNCaP and C4-2 cells treated for 72 h with SAL (*n* = 3). **B** Induction of p-AKT levels in *CDKN2B*-knockdown cells. Numbers indicate mean ± SD. **C** Detection of SA-β-Gal activity of LNCaP and C4-2 cells incubated for 72 h with DMSO, LAL, or SAL in combination with or without AKT inhibitor (AKTi). Bars show means ± SD (*n* = 3). **D** Analyses of mRNA level of *CDKN2B* by the indicated treatments in LNCaP and C4-2 cells. Expression was normalized to the housekeeping genes *TBP* and *GAPDH* (*n* = 3). **E** Detection of p15 protein level. **F** Detection of p-AKT and its downstream target p-S6 (*n* = 3). **p ≤ 0.01, ***p ≤ 0.001, ****p ≤ 0.0001.

The AR is known to interact with AKT/PKB, leading to its phosphorylation at a nongenomic level by rapid signaling. The androgen-induced phosphorylation events are suggested to disappear within one hour of treatment [19, 21–23]. However, our data suggest that SAL treatment enhanced phosphorylation of AKT in both LNCaP and C4-2 cells also in a long-term manner being detectable after 72 h of treatment (Fig. 3A). The underlying data indicate a persistent long-term nongenomic AR activity. Knockdown of p15^INK4b^ did not repress SAL-induced phosphorylation of AKT (Fig. 3B), revealing that the AR-AKT interaction is upstream of p15^INK4b^ in the SAL-induced senescence pathway.

Interestingly, cotreatment of cells with SAL and AKTi resulted in reduction of cellular senescence levels in both cell lines (Fig. 3C). This reveals that SAL-induced cellular senescence is mediated through the AR-AKT interaction. Accordingly, the SAL-induced *CDNK2B* mRNA and p15^INK4b^ protein levels were reduced by AKTi (Fig. 3D, E). The data confirm that AKT signaling is part of the SAL-induced cellular senescence in both CSPC and CRPC cells being upstream of p15^INK4b^. Phosphorylation of AKT at S473 (p-AKT) activates AKT signaling [24]. In line with this, the level of the AKT downstream target, phospho-S6 (p-S6), is enhanced by SAL (Fig. 3F). The treatment with AKTi reduced the SAL-induced p-S6 levels, more pronounced in C4-2 cells, while only slightly reducing p-AKT level. These data suggest that after three days of treatment with SAL, androgen induces a nongenomic AR-AKT long-term interaction.

**Fig. 4:**
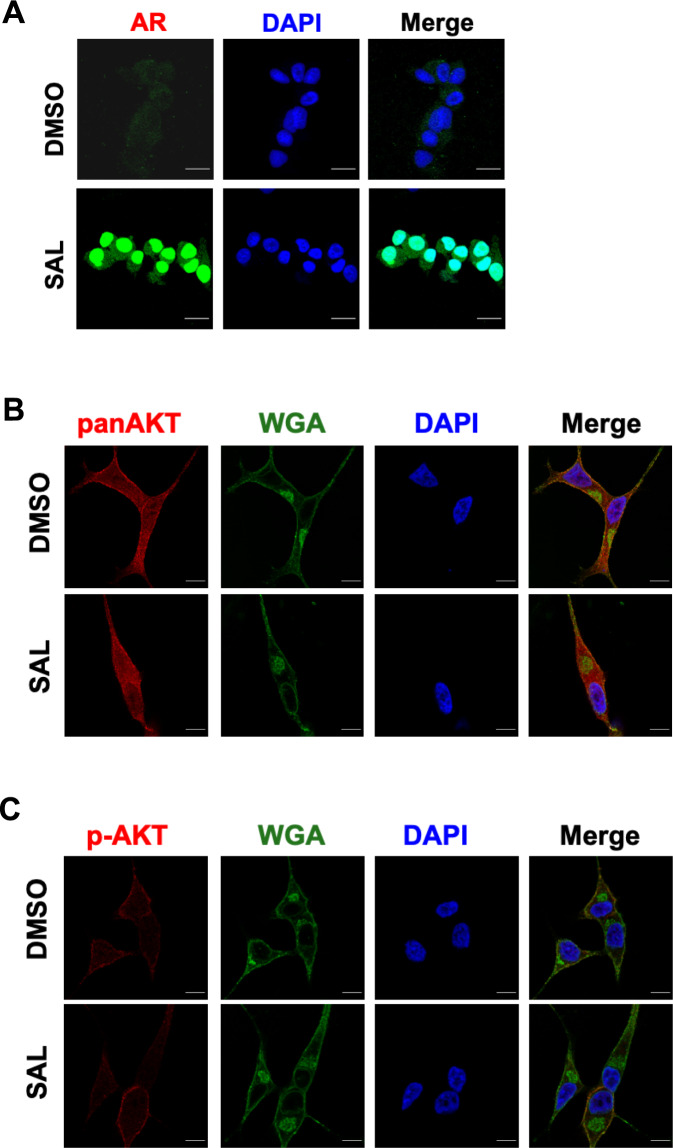

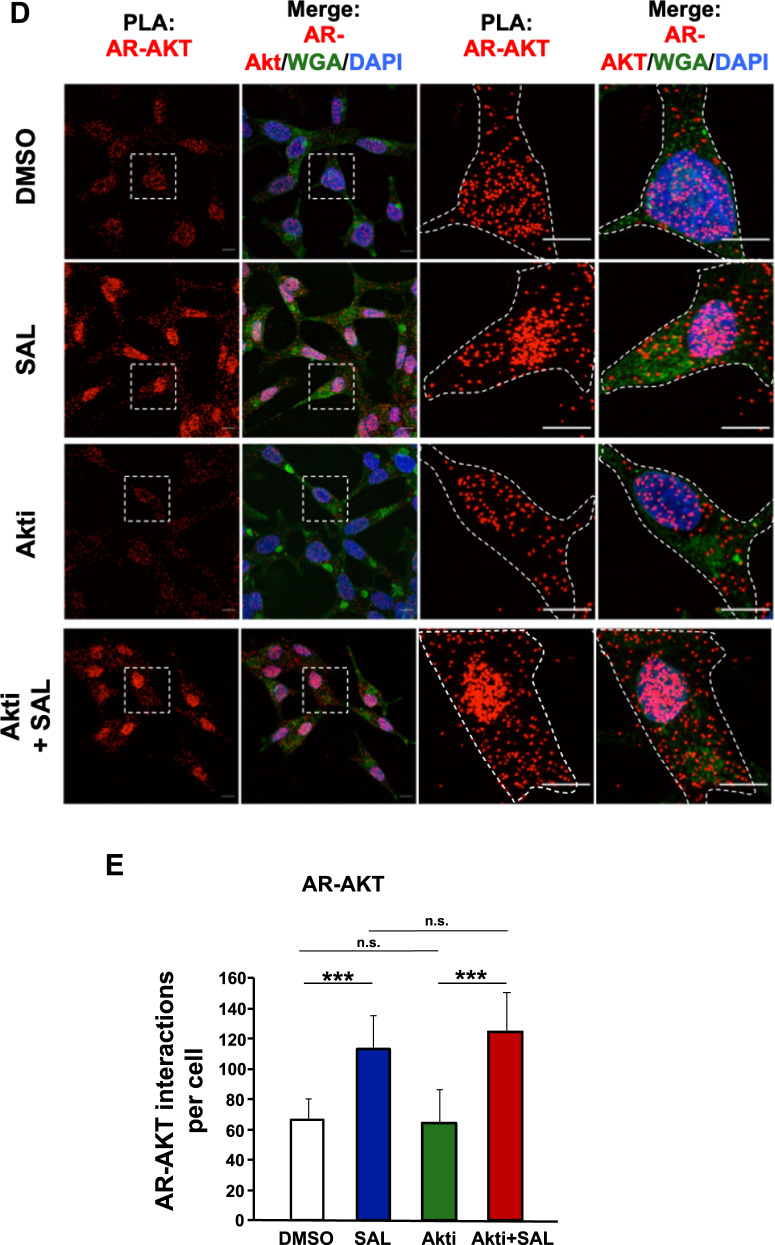
SAL promotes interaction of AR with AKT. **A** Immunofluorescence detection to visualize intracellular localization of AR (green) in LNCaP cells by high-resolution confocal scanning fluorescence microscopy. Nuclei are stained by DAPI (blue). Scale bar indicates 2 µm. **B**, **C** Intracellular detection of AKT (**B**) and p-AKT (**C**) by superresolution confocal scanning fluorescence microscopy (red). Wheat germ agglutinin (WGA) was used as membrane marker (green). Scale bar indicates 2 µm (*n* = 3). **D** Quantitative proximity-ligation assays (PLA) were performed to analyze native intracellular interaction of endogenous AR with endogenous AKT in the presence of SAL and AKTi. Cells were treated for 72 h. Shown are representative pictures. Scale bar indicates 10 µm. **E** The number of PLA signals per cell was counted by Fiji software. PLA signals were calculated from 80 cells derived from three independent experiments. Bar graphs are shown as mean ± SEM, ***p ≤ 0.001, n.s. not significant.

To test the hypothesis that SAL promotes the interaction of AR with AKT, we used laser-scanning high-resolution microscopy. As expected, the endogenously expressed AR is localized predominantly in the cytosol, while SAL treatment enhances its nuclear localization (Fig. 4A). AKT was detected at the cell membrane, in speckles in the cytoplasm, as well as in the nucleus (Fig. 4B). This distribution was independent of SAL treatment. Wheat germ agglutinin (WGA) was used to detect cellular membranes [25]. p-AKT was preferentially detected at the plasma membrane and was also found in the cytosol (Fig. 4C). To analyze whether SAL has an influence on the endogenous interaction of AKT with AR, quantitative proximity-ligation assays (PLA) were performed with detection by high-resolution laser-scanning microscopy (Fig. 4D). Each dot represents an interaction of AR with endogenous AKT in a protein complex. The data imply that AKT interacts with AR in the cytoplasm as well as in the cell nucleus (Fig. 4D). Quantification of the detected signals indicated an enhanced cytoplasmic and nuclear interaction of AKT with AR by SAL treatment (Fig. 4E). Interestingly, treatment with AKTi did not reduce the AR-AKT interaction (Fig. 4D).

These data demonstrate that the interaction of AKT with AR is not limited to the cytoplasm but rather also occurs in the cell nucleus and suggests that the AR-AKT interaction itself does not correlate with the induction of cellular senescence. Thus, SAL enhanced the level of endogenous AR-AKT interaction detected after three days of treatment.

### Identifying specific senescence transcriptomes induced by SAL and counteracted by AKTi

**Fig. 5:**
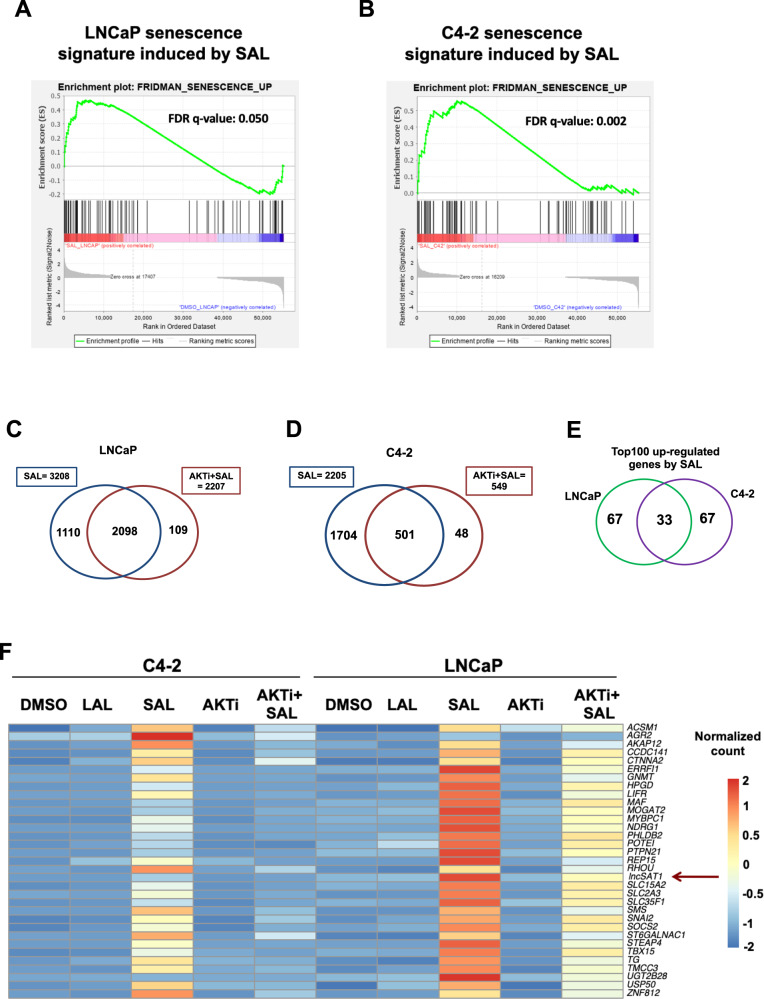

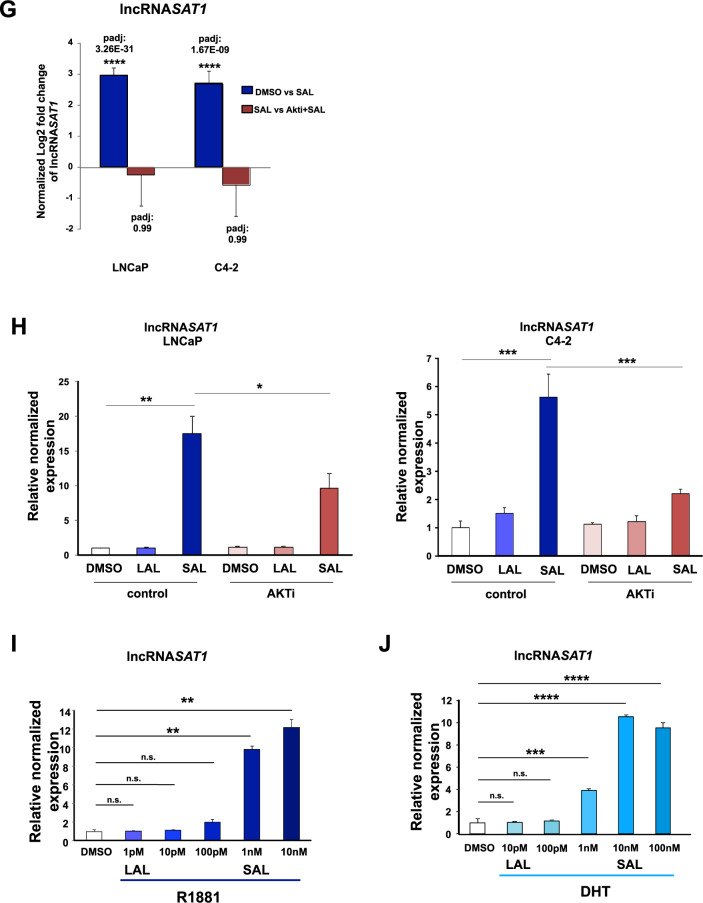
Differential expression analysis indicates that AKTi significantly alters expression of most genes regulated by SAL and identifies the lncRNA*SAT1*. RNA-seq experiments with both cell lines LNCaP and C4-2 were performed with the indicated treatments for 72 h. Data are presented as means ± LFC SE (Log2 fold change standard error). Two-tailed paired Student’s *t*-test was performed for statistical analysis (*n* = 3; ***padj ≤ 0.001, ****padj ≤ 0.0001). **A**, **B** Plot of gene-set enrichment analysis (GSEA) of the senescence. The senescence signature is induced by SAL in both cell lines. Positive enrichment of the gene set in R1881 vs. DMSO; in (**A**) LNCaP, FDR *q*-value = 0.050, and (**B**) C4-2, FDR *q*-value = 0.002. **C**, **D** GSEA plot for senescence. Venn diagram depicts the number of genes being significantly regulated by SAL and number of genes altered by AKTi cotreatment in (**C**) LNCaP or (**D**) C4-2 cells. The induced senescence signature is significantly rescued by AKTi in both (**A**) LNCaP, FDR *q*-value = 0.002 and (**B**) C4-2 cells, FDR q-value=0.083. **E** Venn diagram indicates the overlap of top100 significantly SAL-regulated genes between LNCaP and C4-2 cells. **F** Heat map represents the 33 genes upregulated upon SAL in both LNCaP and C4-2 cells. Color-key number represents normalized count. **G** Normalized log_2_ fold change of lncRNA*SAT1* upon SAL or AKTi+SAL in both LNCaP and C4-2 cells of RNA-seq data. **H** Confirmed regulation of the lncRNA*SAT1* using qRT-PCR in both LNCaP and C4-2 cells. **I**, **J** Concentration-dependent induction of the lncRNA*SAT1* by androgens using (**I**) R1881 or (**J**) DHT in LNCAP cells treated for 72 h by qRT-PCR. Values were normalized to *TBP* and *GAPDH* and relative expression was calculated compared with control treatment. Bars indicate the mean ± SEM (*n* = 4). *p ≤ 0.05, **p ≤ 0.01, ***p ≤ 0.001, ****p ≤ 0.0001, n.s. not significant.

In order to reveal the impact of AR-AKT signaling to induce SAL-mediated cellular senescence in PCa cells at the transcriptome, RNA-seq experiments were performed with both cell lines. Our hypothesis was that SAL-mediated pathways to induce cellular senescence include gene sets upregulated by SAL and being downregulated by AKTi in the presence of SAL in both cell lines. Accordingly, this coregulated gene set may contain factors mediating SAL-induced cell senescence. To identify transcriptome landscapes, RNA deep sequencing was performed with both LNCaP and C4-2 cells with and without SAL for three days. In addition, each cell line was either cotreated with SAL and AKTi or treated with AKTi alone for three days. Three independent experiments were performed. Gene-set enrichment analysis (GSEA) shows a positive enrichment of the senescent gene set (FRIDMAN_SENESCENCE_UP) by SAL compared with control (DMSO), indicating a significant induction of senescence signature in both cell lines by SAL (Fig. 5A, B). Further subsequent bioinformatic transcriptome analyses revealed that SAL regulates the expression (repressed or activated) of more than 3100 genes in LNCaP and 2200 genes in C4-2 cells (Fig. 5C, D). Interestingly, the SAL-induced senescence signature is rescued significantly by Akti in both LNCaP and C4-2 cells (Fig. 5C, D) suggesting that surprisingly, a major fraction of these SAL-regulated genes are significantly coregulated by AKT in both cell lines (Fig. 5C, D). These genes include *CDKN2B* (encoding p15^INK4b^), *KLK3* encoding the diagnostic marker PSA, and *TMPRSS2* and *FKBP5* as AR target genes (Supplemental Fig. S1), which also serve as a control for the RNA-seq analyses. Thus, the data suggest that the AR-AKT interaction influences potently the transcriptomics and indicates that the AR-AKT signaling has, in addition to a rapid, nongenomic activity, a wide genomic signaling when treated for three days.

Analyzing the overlap of significantly upregulated top-100 genes by SAL in both cell lines, it revealed a gene set of 33 genes (Fig. 5E) that were commonly downregulated by AKTi in the presence of SAL in both cell lines. Thus, the listed gene set meets most of our expectation in order to be upregulated potently and significantly only by SAL and to be inhibited specifically by the combination of SAL plus AKTi in both cell lines. The common upregulation of these genes was lost in C4-2 and strongly reduced in LNCaP cells by AKTi treatment (Fig. 5F). One of the factors being potently upregulated by SAL and counteracted by the cotreatment with AKTi is the lncRNA*SAT1* (Fig. 5G). The RNA-seq data were confirmed by qRT-PCR in both cell lines (Fig. 5H). The lncRNA*SAT1* was selected for further analysis, since the role of lncRNAs in inducing cellular senescence upon SAL treatment is poorly investigated and remains largely unknown. Interestingly, the lncRNA*SAT1* has been reported to act as tumor suppressor in melanoma by suppressing invasion and metastasis. These observations indicate that the lncRNA*SAT1* might play an important role in PCa.

Using the AR agonists, R1881 or DHT, a concentration-dependent treatment of LNCaP cells was performed. The data show a potent upregulation of lncRNA*SAT1* by SAL when using 1 nM R1881 or 10 nM DHT (Fig. 5I, J). Notably, the expression of the lncRNA*SAT1* is reduced by AR antagonists (supplemental Fig. S2).

### The lncRNA*SAT1* is induced by SAL in PCa cells and tumor samples treated ex vivo

**Fig. 6:**
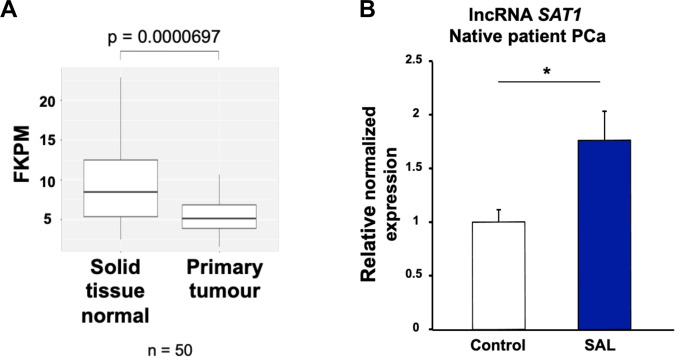
The expression of lncRNA*SAT1* is reduced in PCa and is inducible by SAL in tumor tissues. **A** TCGA database of 50 patient PCa samples, comparing the expression in nontumor and tumor areas, indicates that the expression of lncRNA*SAT1* is lower in primary tumor compared with normal tissues with a highly significant difference. **B** Human PCa tissues obtained from prostatectomy were treated with SAL ex vivo using 1 μM R1881. Analysis of the gene expression of lncRNA*SAT1* was performed by qRT-PCR. Values were normalized to the housekeeping gene *TBP* and indicated with the mean ± SEM (*n* = 7).

Analyzing the cancer genome atlas (TCGA) database, the expression of lncRNA*SAT1* was evaluated in 50 samples of PCa patients comparing primary tumor with normal prostate tissue samples. The expression of lncRNA*SAT1* is significantly lower in primary tumor compared with normal tissues (Fig. 6A). Comparing different Gleason scores revealed no significant change of lncRNA*SAT1* expression (data not shown). Thus, the data suggest a downregulation of lncRNA*SAT1* in PCa tumor samples compared with nontumor tissue. To reveal whether the expression of lncRNA*SAT1* is regulated by SAL in PCa patient samples, native tumor samples obtained from patients with radical prostatectomy were treated ex vivo with SAL. The data indicate that the expression of the lncRNA*SAT1* is upregulated by SAL in patient’s samples (Fig. 6B), which is in agreement with the data from LNCaP and C4-2 cell lines.

### The lncRNA*SAT1* mediates SAL-induced cellular senescence

**Fig. 7:**
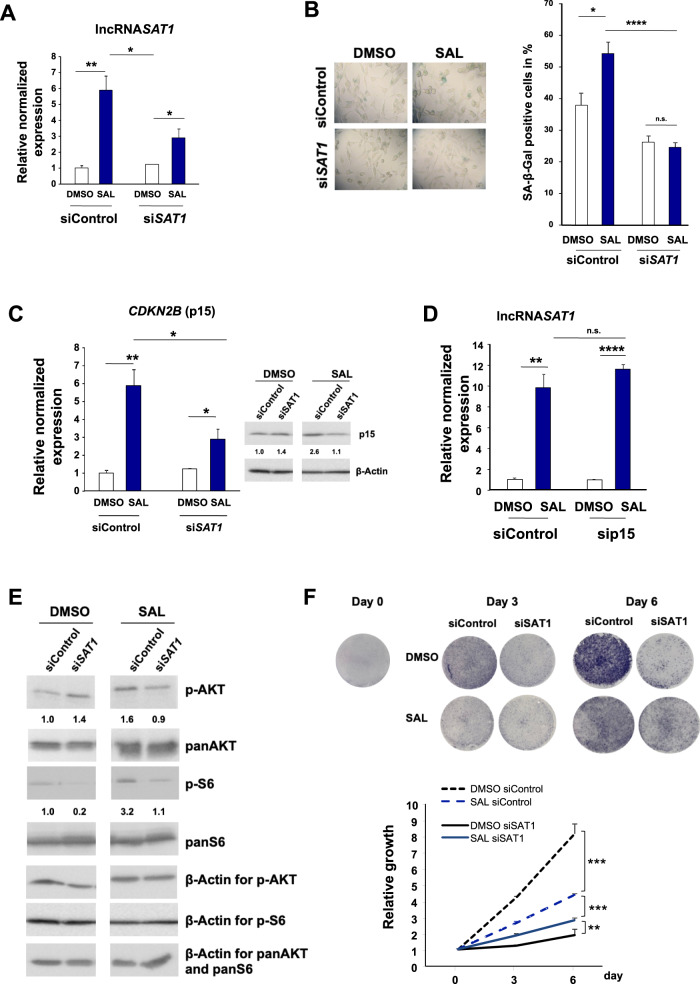

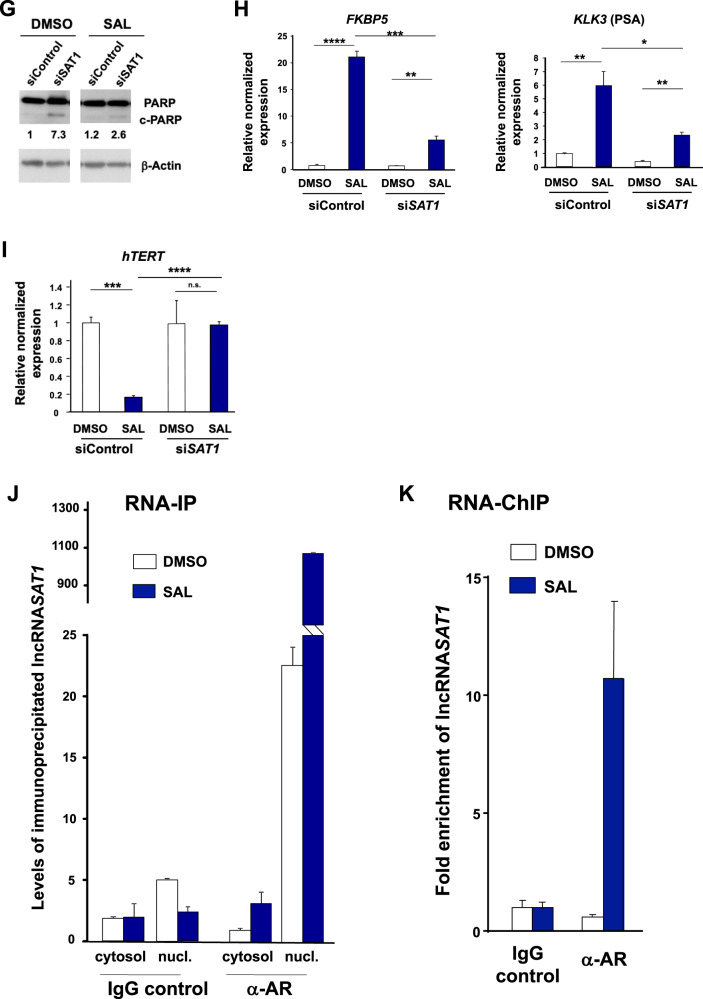
The lncRNASAT1 regulates *CDKN2B*/p15 and the Akt signaling to induce cellular senescence and control PCa growth. LNCaP cells were transfected with nontargeting siRNA (siControl) as negative control or si*SAT1* targeting the additional exon in lncRNA*SAT1*. After 24 h of transfection, cells were treated with SAL or 0.1% DMSO as solvent control for 48 h followed by RNA extraction. **A** The knockdown of lncRNA*SAT1* in LNCaP cells was confirmed with qRT-PCR (*n* = 3). **B** The level of cellular senescence was analyzed by quantification of SA-β-Gal- positive stained LNCaP cells (*n* = 3). **C** The levels of *CDKN2B* mRNA and p15^INK4B^ protein were analyzed with and without lncRNA*SAT1* knockdown (*n* = 3). **D** The level of lncRNA*SAT1* was analyzed in the *CDKN2B-*knockdown LNCaP cells using qRT-PCR (*n* = 3). **E** Changes of panAKT, p-AKT, panS6, and p-S6 levels after knockdown of lncRNA*SAT1* (*n* = 3). **F** Growth curves of LNCaP cells with and without SAL treatment comparing with and without knockdown of lncRNA*SAT1* analyzed by crystal violet staining (*n* = 2). **G** LncRNA*SAT1* suppression mediates apoptosis in LNCaP cells. Western blot analysis of cleaved PARP in response to lncRNA*SAT1* knockdown after 48 h of treatment with DMSO as solvent control or 1 nM R1881 (SAL). The protein expression was normalized to the loading control β-Actin using LabImage 1D software (*n* = 2). **H**, **I** qRT-PCR was used to analyze the expression of AR target genes through knockdown of lncRNA*SAT1* in LNCAP cells treated with and without SAL. Relative expression was calculated compared with solvent control. The mean ± SEM values were calculated from three independent experiments (*n* = 3). Indicated are (**H**) the positively androgen-regulated *KLK3* and *FKBP5* genes and (**I**) the negatively androgen-repressed *hTERT* gene. **J** LNCaP cells were incubated for 24 h with DMSO or SAL followed by cytosolic and nuclear extraction. Immunoprecipitation was performed using an AR antibody or a matched immunoglobulin G (IgG) as control. RNA-immunoprecipitated lncRNA*SAT1* was analyzed by qRT-PCR (*n* = 2). **K** RNA-ChIP was performed with cross-linked chromatin from LNCaP cells treated with DMSO or SAL for 24 h followed by immunoprecipitation of AR and qRT-PCR detection of the lncRNA*SAT1* (*n* = 4). *p ≤ 0.05, **p ≤ 0.01, ***p ≤ 0.001, ****p ≤ 0.0001, n.s. not significant.

To analyze whether the lncRNA*SAT1* is part of SAL-mediated cellular senescence, knockdown experiments were performed using siRNA in LNCaP cells (Fig. 7A). The SAL-mediated induction of cellular senescence was potently inhibited by the lncRNA*SAT1* knockdown (Fig. 7B). This suggests that the lncRNA*SAT1* mediates SAL-induced cellular senescence and indicates that lncRNA*SAT1* is within the AR-mediated pathway of cellular senescence.

To analyze whether the lncRNA*SAT1* is upstream or downstream of SAL-induced p15^INK4b^, the expression of *CDKN2B* was analyzed (Fig. 7C). Both p15^INK4b^ mRNA and protein levels were reduced by knockdown of lncRNA*SAT1* upon SAL suggesting that the lncRNA*SAT1* is upstream of *CDKN2B* expression. The knockdown of p15^INK4b^, on the other hand, does not affect the SAL-dependent lncRNA*SAT1* upregulation (Fig. 7D), further indicating that the lncRNA*SAT1* is an upstream factor of p15^INK4b^ in SAL-mediated cellular-senescence pathway.

In addition, the knockdown of lncRNA*SAT1* reduces the level of p-AKT and its downstream target p-S6 upon SAL treatment (Fig. 7E). This indicates that the lncRNA*SAT1* is also upstream of the p-AKT signaling. Thus, the data suggest a novel AR feedback loop to induce cellular senescence by enhancing the expression of the lncRNA*SAT1*-AKT and p15^INK4b^.

Because SAL-induced cellular senescence is known to arrest cell cycle [4], we analyzed the growth properties of lncRNA*SAT1*-knockdown cells (Fig. 7F). The knockdown of lncRNA*SAT1* reduces potently the growth of LNCaP cells in the absence of SAL. However, in the presence of SAL the cell proliferation is higher in knockdown cells compared with DMSO control (Fig. 7F). This indicates that the knockdown of lncRNA*SAT1* has a dual activity dependent on the presence of SAL. We observed an enhanced apoptosis in knockdown cells in the presence of DMSO treatment, which may account for the reduced cell proliferation. In line with the growth properties in the presence of SAL, the cleaved PARP marker is reduced by the lncRNA*SAT1* knockdown (Fig. 7G). These data suggest that the lncRNA*SAT1* reduces apoptosis, perhaps by influencing the AR-AKT signaling, since the AKT pathway is known as a prosurvival pathway [26].

### The lncRNA*SAT1* is an AR coregulator

These data led to the hypothesis that the lncRNA*SAT1* might interact with the AR and influences the AR transcriptional activity. First, we analyzed the expression of FKBP5- and PSA-encoding genes as directly regulated AR target genes by knocking down lncRNA*SAT1*. The expression of both *FKBP5* and *KLK3*, encoding PSA, was potently upregulated by SAL and downregulated by the knockdown of lncRNA*SAT1* (Fig. 7H). This effect suggests that the lncRNA*SAT1* is a coactivator of the AR. On the other hand, the repression of the AR-downregulated target gene *hTERT*, encoding the catalytic subunit of the human telomerase, is abolished by the knockdown of lncRNA*SAT1* (Fig. 7I). This indicates that the lncRNA*SAT1* mediates at least in part AR-mediated repression of the *hTERT* gene.

Thus, the data suggest that lncRNA*SAT1* is interacting with AR as a coregulator.

### The lncRNA*SAT1* interacts with AR

Based on these data, we hypothesized that the lncRNA*SAT1* might bind to AR as a coregulator. To address this, we immunoprecipitated the AR from SAL-treated cells and analyzed by qRT-PCR whether the lncRNA*SAT1* is co-immunoprecipitated (RNA-Co-IP) (Fig. 7J). In addition, we analyzed the preferred intracellular interaction of the lncRNA*SAT1* with AR and separated the cytosolic fraction from nuclear fraction. Interestingly, we detected specific binding of the lncRNA*SAT1* to AR in both cytosolic and nuclear fractions (Fig. 7J). Higher levels of immunoprecipitated lncRNA*SAT1* were detected in the nucleus, which may be due to higher nuclear AR concentrations by SAL (supplemented Fig. S3) and/or by association with chromatin. To address this, RNA-ChIP experiments were performed. The interaction was confirmed by chromatin immunoprecipitation of AR and detection of the lncRNA*SAT1* by qRT-PCR in the immunoprecipitated chromatin isolates using anti-AR antibody (Fig. 7K). An enhanced level of lncRNA*SAT1*was detected in the presence of SAL (Fig. 7K), indicating that the lncRNA*SAT1* interacts with AR on chromatin. Thus, these data suggest that the lncRNA*SAT1* binds to AR in the cytosol and nucleus. The interaction is enhanced by treatment with SAL and occurs also on chromatin. Whether it is a direct or indirect binding is unclear. However, using the data calculation by http://pridb.gdcb.iastate.edu/RPISeq, high interaction probabilities between lncRNA*SAT1* and AR protein were predicted with a high score: RF classifier: 0.8 and SVM classifier: 0.98, supporting our data (supplemental Fig. S4).

**Fig. 8:**
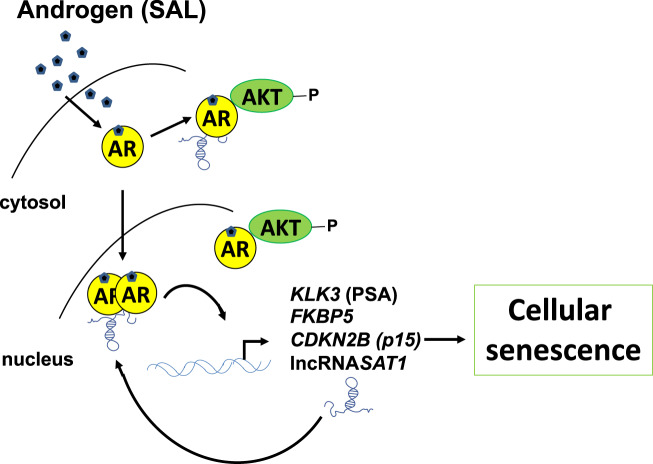
Schematic view of the AR-lncRNA*SAT1*-AKT-p15 axis. SAL treatment induces the AR-AKT interaction and AKT phosphorylation, induction of p15, and cellular senescence. The knockdown of the lncRNA*SAT1* reduces AKT phosphorylation, the expression of the AR target genes encoding PSA and FKBP5, as well as p15^INK4B^ protein level, leading to reduced level of cellular senescence. Further, the knockdown of p15^INK4B^ reduces the SAL-induced cellular senescence.

Taken together under SAL conditions, the lncRNA*SAT1* binds to AR, upregulates the level of p-AKT and p15^INK4b^ to control cellular senescence and AR signaling at both the nongenomic and genomic level. Thus, we propose a novel regulatory axis of AR in PCa cells involving the lncRNA*SAT1* that regulates AR-mediated transactivation, AKT signaling, and cellular senescence (Fig. 8).

## Discussion

Previous findings indicated that SAL induces cellular senescence in LNCaP cells, an irreversible cell cycle arrest and thus an important tumor-suppressive mechanism [4].

In addition to the classical clinical treatment using androgen-deprivation therapy, an approach combining androgen ablation with intermittent cycles of pharmacological SAL, termed BAT was developed [9, 11, 27]. With BAT, CRPC patients are maintained on ADT with luteinizing hormone-releasing hormone (LHRH) and receive cycles of testosterone at supraphysiological level. A radiographic response in 50% of metastatic CRPC patients in the first clinical trial was reported. Further, it is suggested that BAT may restore ADT sensitivity in PC patients [11].

Mechanistically, since SAL-meditated cellular senescence was inhibited by the AKT inhibitor AKTi, it suggests that the induction of cellular senescence is at least in part mediated through nongenomic signaling of the AR-AKT pathway [4]. Nongenomic effects were shown to occur rapidly within minutes and disappear shortly after treatment [28]. Here, we addressed a long-term effect of AKT signaling with three days of SAL treatment. It is well known that the AR, as a ligand-dependent transcription factor, regulates gene expression of target genes. These genomic actions have been extensively studied. In addition to the genomic pathway, AR can also activate intracellular signaling cascades by nongenomic actions. In contrast to the genomic actions, nongenomic effects occur within seconds to minutes of androgen treatment, including the activation of the PI3K-AKT pathway [21, 29]. An accumulation of p-AKT was rapidly detected within 5–10 min of androgen treatment that declined after 30–60 min [28]. So far, it has been assumed that these phosphorylation events caused by nongenomic AR signaling disappear within one hour after androgen treatment [19]. However, long-term effects of androgens on AKT and its downstream targets are less examined.

Our data suggest that androgens can activate the AKT-S6 pathway not only within the rapid signaling period but also after long-term androgen treatment. This long-term effect of AR was also confirmed by immunofluorescence staining. After 72 h of SAL treatment, a fraction of AR was still clearly detectable in the cytoplasm. The cytoplasmic AR can mediate nongenomic actions such as the phosphorylation of AKT [28]. Thus, the data indicate that the nongenomic AR signaling in the cytoplasm is not restricted to a short-time period, rather the signaling lasts longer than yet assumed. Therefore, this study provides new insights into the long-term effects of androgen signaling that might be useful to explain some effects of SAL on the growth of PCa.

Interestingly, our data further indicate that AR directly interacts with AKT in the cytoplasm and nucleus. In addition, SAL treatment increased the AR-AKT interaction in both cellular compartments after 72 h of hormone treatment. The interaction between AR and AKT may lead to AKT activation and recruitment of other proteins to this complex. These data suggest that after three days of treatment with SAL, androgens are able to induce a long-term AR-AKT interaction.

A previous report indicated by co-immunoprecipitation experiments that AR and AKT interact predominantly in lipid rafts of LNCaP cells after short-term androgen treatment for 1 h. Interestingly, low levels of AR-AKT complexes were also detected in cytoplasmic and nuclear fractions [28]. Therefore, it is possible that after longer treatment with SAL, the AR-AKT interactions dislocate from the lipid rafts to the cytoplasm and the nucleus.

Surprisingly, AKTi did not affect the AR-AKT interaction. However, AKTi in combination with SAL inhibited the phosphorylation and inactivation of the AKT downstream targets FOXO3a and TSC2 compared with SAL alone (data not shown). The phosphorylation levels of AKT were not potently reduced by AKTi, suggesting that AKTi rather acts through another mode of action to inactivate AKT. One possible explanation is that AKTi might induce conformational changes in AKT, leading to the inhibition of its catalytic activity and to suppression of AKT downstream targets, observed by Western blot.

In line with our PLA results, the occurrence of AKT in the nuclear compartment was described earlier [30, 31]. However, it is still unclear how AKT translocates into the nucleus since a nuclear-localization signal of AKT is unknown [31]. It is possible that AR is involved in the nuclear localization of AKT by transporting AKT in a complex with AR through the nuclear-pore complex. Several components, such as PIP_3_, PDK1, and mTORC2, that are important for the activation of AKT, are localized in the nucleus, suggesting that AKT might interact with other factors within the cell nucleus [32–34]. It has been demonstrated that nuclear AKT phosphorylates acinus, resulting in its resistance to caspase cleavage and inhibition of apoptotic chromatin condensation [31, 35]. Since apoptosis resistance is a main feature of senescent cells, the nuclear AR-AKT complex might be involved in processes regulating cellular senescence. However, the effect of the AR-AKT complex formation on these processes has yet to be investigated. Thus, it is also conceivable that nuclear AR-AKT complexes not only activate nongenomic pathways but also have genomic effects.

In conclusion, the data indicate that the interaction of AR with AKT is not limited to the plasma membrane or to the cytoplasm, but also takes place in the nucleus.

In recent years, many lncRNAs have been implicated in various cancers, including PCa [36–38]. The lncRNA*SAT1* was identified by RNA sequencing as potential gene that could be involved in cellular-senescence induction by SAL and therefore might play an important role in PCa.

The lncRNA*SAT1* was upregulated ex vivo in human PCa tissues by androgen treatment and revealed a concentration-dependent inducibility in LNCaP cells, suggesting a tumor-suppressive function. Interestingly, the cancer genome atlas (TCGA) database indicates that the expression of lncRNA*SAT1* is downregulated in primary PCa tumor tissue compared with nontumor tissue of the same patient. In line with this, knockdown of lncRNA*SAT1* suppressed the SAL-induced cellular senescence. Further, the knockdown of lncRNA*SAT1* downregulated p15^INK4b^ both at protein and mRNA expression upon SAL, suggesting that lncRNA*SAT1* is an upstream regulator of p15^INK4b^. Previously, we revealed upregulation of p16^INK4a^ upon SAL treatment, a known senescence-inducing factor. Similar to p16^INK4a^, the tumor suppressor p15^INK4b^ belongs to the CDK-inhibitor family that binds CDK4/6. Thus, in addition to p16^INK4a^ also p15^INK4b^ is induced upon SAL treatment, suggesting that the cell cycle inhibitors encoded by the INK locus regulate androgen-induced cellular senescence. Thereby, the phosphorylation of pRb is inhibited, leading to cellular-senescence initiation [39, 40]. Indeed, hypophosphorylation of pRb was observed upon SAL treatment [4]. Consequently, lncRNA*SAT1* might mediate cellular senescence by the induction of p15^INK4b^ expression. Moreover, knockdown of lncRNA*SAT1* suppressed phosphorylation of AKT at S473 upon SAL treatment, suggesting that the lncRNA*SAT1* signaling is upstream of AKT for SAL-induced cellular senescence.

Recent studies demonstrate the potential role of lncRNAs as drivers in tumor-suppressive or oncogenic pathways [36]. In this study, it was observed that knockdown of lncRNA*SAT1* significantly suppressed the growth of LNCaP cells in the absence of SAL. However, proliferation was enhanced in knockdown cells treated with SAL. This suggests that lncRNA*SAT1* has a dual activity, depending on the androgen level. The knockdown of lncRNA*SAT1* led to the induction of apoptosis, indicating that lncRNA*SAT1* is important for cell survival in the absence of SAL.

In response to SAL, the induction of apoptosis by si*SAT1* was strongly reduced. This leads to the hypothesis that lncRNA*SAT1* is important for the induction of cellular senescence in cells that are resistant to apoptosis. The hypothesis is strengthened by the observation that AKT suppresses the androgen-induced apoptosis [22]. Here, it was shown that lncRNA*SAT1* promotes AKT signaling and therefore the AKT-mediated prosurvival effects. Yet, it suggests that the lncRNA*SAT1* is crucial for the induction of senescence upon SAL treatment. The tumor-suppressive function of lncRNA*SAT1* is in line with a publication showing that knockdown of lncRNA*SAT1* promotes proliferation, invasion, and metastasis of melanoma cells [41]. Our results confirm that lncRNA*SAT1* might act as a tumor suppressor induced by SAL and therefore could serve as a novel therapeutic target for the treatment of PCa.

Since the expression of the lncRNA*SAT1* is induced by SAL and its knockdown reduces the levels of p-AKT and p15^INK4b^ and the level of cellular senescence, it suggests a positive feedback loop in AR-mediated cellular senescence (Fig. 8). In conclusion, a novel regulatory axis with the hierarchy of AR, lncRNA*SAT1*, AKT, and p15^INK4b^ was identified, which is important for the induction of cellular senescence in PCa cells. Taken together, all these results shown here may help to understand the molecular processes that take place during BAT.

## Materials and methods

### Cell culture and ligand treatment

LNCaP and C4-2 cell lines were regularly tested for mycoplasmas and cultured as described previously [4]. We used 1 nM R1881 or 10 nM DHT as SAL and DMSO as solvent control. To knockdown lncRNA*SAT1* and p15^INK4b^, cells were transfected with ON-TARGETplus Human *SAT1* siRNA individually targeting the additional exon of lncRNA*SAT1* or ON-TARGETplus Human CDKN2B siRNA (set of 4) with a final concentration of 25 nM in each well. As a negative control, ON-TARGETplus nontargeting siRNA was used. All siRNAs were transfected by the DharmaFECT reagent (Dharmacon) according to the manufacturer’s protocol. Transfection was performed 24 h prior R1881 treatment. Thereafter, cells were incubated for 48 h with androgen. Growth assays were described previously [4] using crystal violet staining.

ON-TARGETplus Human *SAT1* siRNA:

sense 5′-CAUGAUAAAUGAGGACACAUU-3′;

antisense 5′-UGUGUCCUCAUUUAUCAUGUU-3′

For p15^INK4b^ stable-knockdown experiments, pLMP-shLuc served as negative control with pLMP-sh*CDKN2B* as knockdown vector transfected via the GenJET^TM^ in human LNCaP cells generating LNCaP-pLMP-shLuc and pLMP-sh*CDKN2B* sublines, respectively. For selection of transfected cells (stable transfection), 1 µg/µl puromycin was added to the cell culture medium. The sh*CDKN2B* oligonucleotide sequence:

5′-TGCTGTTGACAGTGAGCG**AACTCAGTGCAAACGCCTAGAT**TAGTGAAGCCA CAGATGTA**ATCTAGGCGTTTGCACTGAGTC**TGCCTACTGCCTCGGA – 3′

### RNA sequencing and transcriptome analysis

See supplement for description.

### Senescence-associated beta-galactosidase (SA-β-Gal) staining

For SA-β-Gal staining, 50,000 cells per well in a 6-well plate were seeded. The staining was performed as described previously [4, 42].

### Ex vivo treatment of prostate-cancer samples

The experimental setup was described previously [43]. All patients informed consent and all were informed about the purpose of the study. The study was approved by the Ethics Committee of the Friedrich-Schiller-University (ethical approvals 3286-11/11 and 2019–1502) and it is conformed to the Declaration of Helsinki. The level of lncRNA*SAT1* was determined in patient’s samples with Gleason score 7. Presurgery PSA values were between 7 and 10 ng/ml.

### Reverse-transcription quantitative real-time PCR (qRT-PCR)

was performed as previously described [44]. Primers for lncRNA*SAT1:* fw: CAGTCTCTAGCTTCGCCATGTA, rev: CCAACAATGCTGTGTCCTCAT.

### Antibodies and Western blot analyses

Preparation of whole-cell lysates and Western blotting was performed as described elsewhere ([44], and supplement).

### Quantitative in situ proximity-ligation assay (PLA)

Proximity-ligation assay (PLA) was performed using Duolink™ In Situ Orange Starter Kit Goat/Rabbit according to the manufacturer’s protocol (Sigma Aldrich). The imaging of the slides was performed using Zeiss LSM 880 with Airyscan in superresolution using a Plan-Apochromat 63x/1.4 oil DIC M27 objective confocal scanning fluorescence microscope. The number of PLA signals per cell was counted by semiautomated image analysis from 30 cells for each treatment by using Fiji software. Total cell and nucleus delineation was initiated using DAPI and WGA channels. In situ PLA signals were automatically counted by defining intensity threshold (set for all images constant). The experiment was repeated three times.

### RNA immunoprecipitation (RNA-IP)

LNCaP cells were treated for 24 h with solvent control or SAL. AR (Abcam, ab19066) or IgG (goat IgG, Abcam, ab6885) antibodies were added to protein G Dynabeads (Invitrogen) and incubated at 4 °C for 1 h. Antibody-bound beads were incubated with cytosolic and nuclei extracts for 2 h at 4 °C. After three washing steps on magnetic rack, RNA extraction was performed using peqGOLD TriFast.Glycogen (Invitrogen). cDNA was obtained by High Capacity cDNA reverse transcriptase kit (Invitrogen).

### RNA–chromatin immunoprecipitation (RNA–ChIP)

LNCaP cells were treated with 1 nM R1881 (SAL) or DMSO as solvent control for 24 h. ChIP was performed using the SimpleChIP^®^ Enzymatic Chromatin IP Kit (Magnetic Beads) according to the manufacturer’s protocol (Cell Signaling). Cells were fixed in 1% formaldehyde for 10 min at room temperature. Following cross-linking, chromatin was sheared to a size of 150–900 bp by sonication using the Bioruptor Pico (diagenode) for 10 cycles (30 sec on, 30 sec off). Next, 10 µg of sheared chromatin were incubated with 1 µg of AR antibody (Cell signaling, 5153) or 1 µg of normal rabbit IgG (Cell Signaling, 2729) as a negative control overnight at 4 °C followed by incubation with 30 µl of protein-G magnetic beads for 2 h at 4 °C. After three wash steps, cross-links were reversed and chromatin was incubated with proteinase K. RNA was isolated using TRIzol Reagent (ambion) according to the manufacturer’s protocol. One-step qRT-PCR was conducted using SuperScript III Platinum SYBR Green One-Step qRT-PCR Kit (Invitrogen), specific primers for lncRNA*SAT1*, and Bio-Rad CFX96TM Real Time PCR detection system. The fold enrichment was calculated normalized to input and set relative to IgG as negative control.

### Statistical analysis

Two-tailed unpaired *t*-test was used for the comparison of the mean values between two groups in GraphPad Prism 8.0 software. Two-way ANOVA was used for multiple comparisons. Experiments were repeated at least two times. Mean, standard deviation (SD), and standard error of mean (SEM) were calculated from the number of biological replicates (n). A 95% confidence interval (*p*-value (*p*)<0.05) was considered as statistically significant (*) between two groups. A 99% confidence interval (*p* < 0.01) and a 99.9% confidence interval (*p* < 0.001) were indicated by two (**) and three stars (***), respectively.

## Supplementary information

S1

S2

S3

S4

Supplemental text

## Data Availability

The RNA-sequencing data are available in the gene expression omnibus (GEO) database under the accession numbers GSE162711, GSE155528, and GSE154755.
