# Correction to: The androgen receptor—lncRNASAT1-AKT-p15 axis mediates androgen-induced cellular senescence in prostate cancer cells

**DOI:** 10.1038/s41388-021-02125-5

**Published:** 2021-12-20

**Authors:** Kimia Mirzakhani, Julia Kallenbach, Seyed Mohammad Mahdi Rasa, Federico Ribaudo, Martin Ungelenk, Marzieh Ehsani, Wenrong Gong, Nikolaus Gassler, Mirjam Leeder, Marc-Oliver Grimm, Francesco Neri, Aria Baniahmad

**Affiliations:** 1 Institute of Human Genetics, Jena University Hospital, Jena, Germany; 2 Leibniz Institute on Aging, Jena, Germany; 3 Medical College, Hubei University of Arts and Science, Xiangyang, China; 4 Section of Pathology, Institute of Forensic Medicine, Jena University Hospital, Jena, Germany; 5 Department of Adult and Pediatric Urology, Jena University Hospital, Jena, Germany; 6 Present Address: SCW Medicath LTD, Baolong industrial Town, Shenzhen, China

**Keywords:** Cancer, Prostate cancer

Correction to: *Oncogene* 10.1038/s41388-021-02060-5; Article published online 19 October 2021.

In this article, the figure captions have been incorrectly placed (Figs. 3 and 4) and assigned to the wrong figures (Figs. 5 to 8). Figures 3 to 8 with their captions are given below

**Fig. 3:**
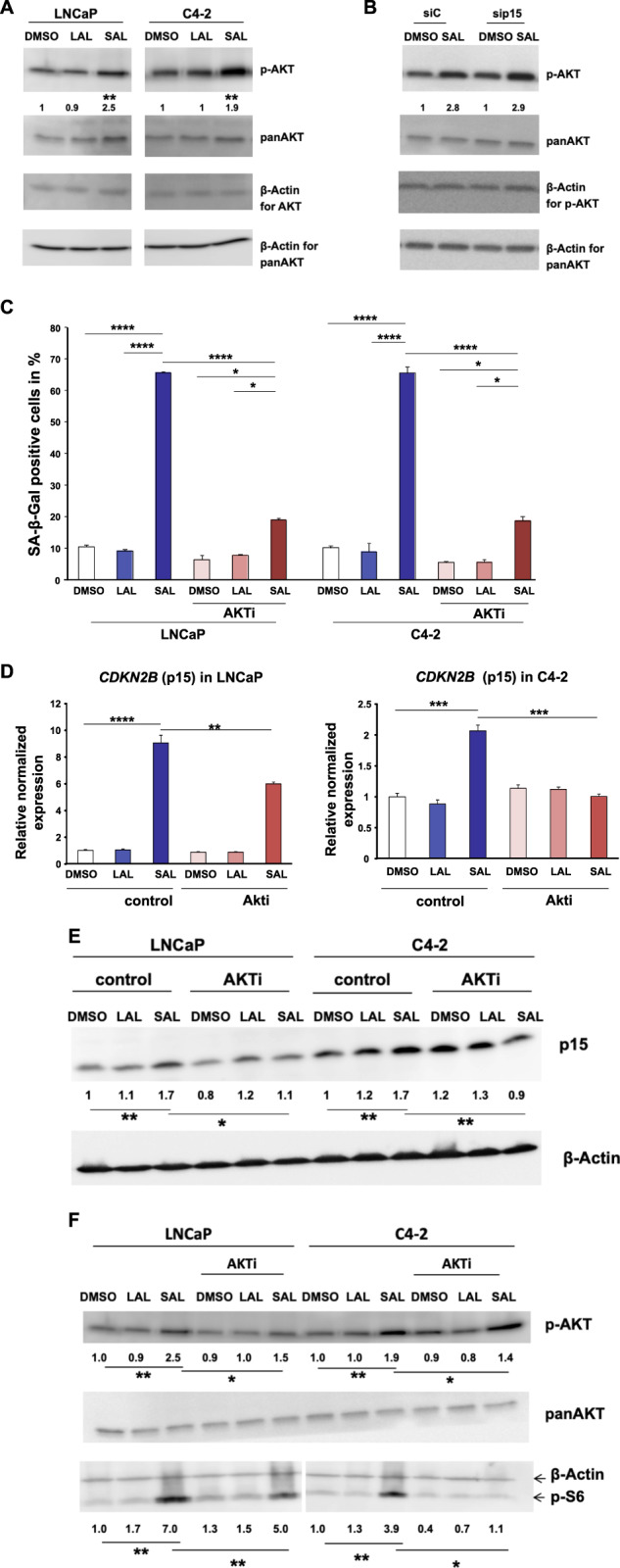
The AKT inhibitor (AKTi) reduces the androgen-induced cellular senescence in both CSPC and CRPC cells. LNCaP cells were treated for 72 h with SAL, LAL, or 0.1% DMSO as solvent control. **A** The level of p-AKT is enhanced in both LNCaP and C4-2 cells treated for 72 h with SAL (*n* = 3). **B** Induction of p-AKT levels in CDKN2B-knockdown cells. Numbers indicate mean ± SD. **C** Detection of SA-β-Gal activity of LNCaP and C4-2 cells incubated for 72 h with DMSO, LAL, or SAL in combination with or without AKT inhibitor (AKTi). Bars show means ± SD (*n* = 3). **D** Analyses of mRNA level of CDKN2B by the indicated treatments in LNCaP and C4-2 cells. Expression was normalized to the housekeeping genes TBP and GAPDH (*n* = 3). **E** Detection of p15 protein level. **F** Detection of p-AKT and its downstream target p-S6 (*n* = 3). **p ≤ 0.01, ***p ≤ 0.001, ****p ≤ 0.0001.

**Fig. 4:**
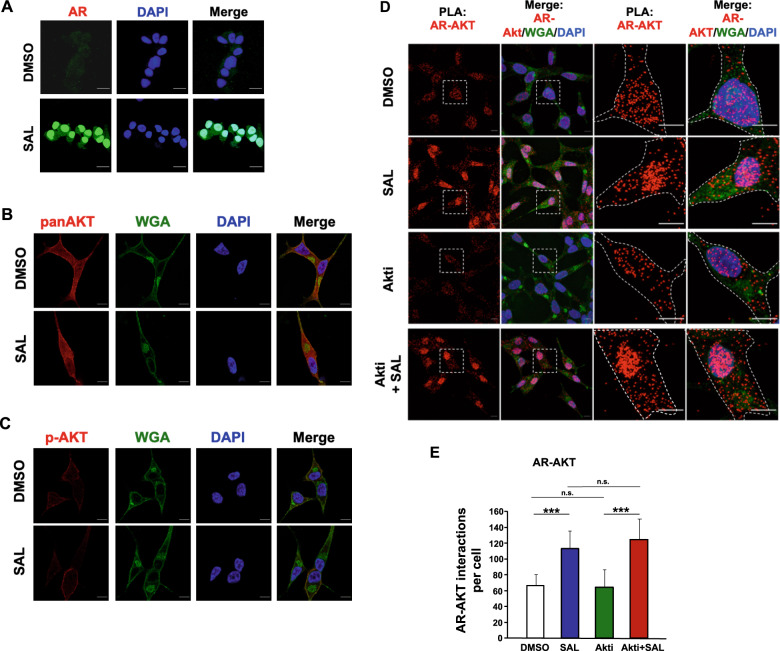
SAL promotes interaction of AR with AKT. **A** Immunofluorescence detection to visualize intracellular localization of AR (green) in LNCaP cells by high-resolution confocal scanning fluorescence microscopy. Nuclei are stained by DAPI (blue). Scale bar indicates 2 μm. **B**, **C** Intracellular detection of AKT (**B**) and p-AKT (**C**) by superresolution confocal scanning fluorescence microscopy (red). Wheat germ agglutinin (WGA) was used as membrane marker (green). Scale bar indicates 2 μm (*n* = 3). **D** Quantitative proximity-ligation assays (PLA) were performed to analyze native intracellular interaction of endogenous AR with endogenous AKT in the presence of SAL and AKTi. Cells were treated for 72 h. Shown are representative pictures. Scale bar indicates 10 μm. **E** The number of PLA signals per cell was counted by Fiji software. PLA signals were calculated from 80 cells derived from three independent experiments. Bar graphs are shown as mean ± SEM, ***p ≤ 0.001, n.s. not significant.

**Fig. 5:**
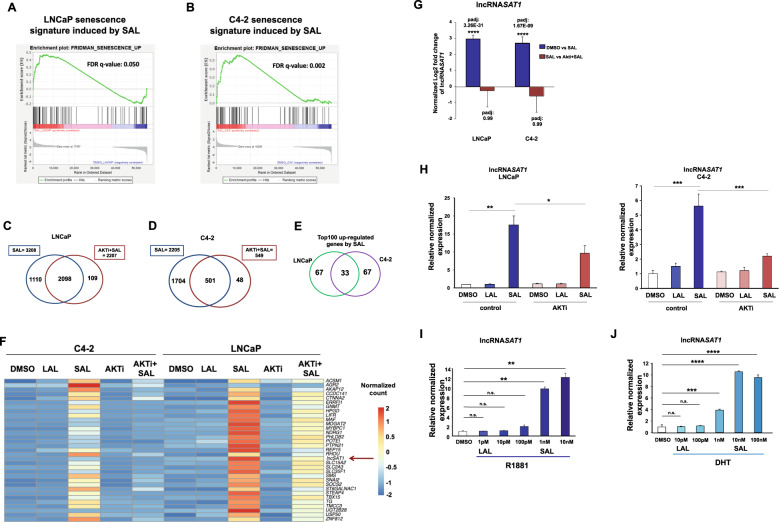
Differential expression analysis indicates that AKTi significantly alters expression of most genes regulated by SAL and identifies the lncRNASAT1. RNA-seq experiments with both cell lines LNCaP and C4-2 were performed with the indicated treatments for 72 h. Data are presented as means ± LFC SE (Log2 fold change standard error). Two-tailed paired Student’s *t* test was performed for statistical analysis (*n* = 3; ***padj ≤ 0.001, ****padj ≤ 0.0001). **A**, **B** Plot of gene-set enrichment analysis (GSEA) of the senescence. The senescence signature is induced by SAL in both cell lines. Positive enrichment of the gene set in R1881 vs. DMSO; in (**A**) LNCaP, FDR *q* value = 0.050, and (**B**) C4-2, FDR *q* value = 0.002. **C**, **D** GSEA plot for senescence. Venn diagram depicts the number of genes being significantly regulated by SAL and number of genes altered by AKTi cotreatment in (**C**) LNCaP or (**D**) C4-2 cells. The induced senescence signature is significantly rescued by AKTi in both (**A**) LNCaP, FDR *q* value = 0.002 and (**B**) C4-2 cells, FDR *q* value = 0.083. **E** Venn diagram indicates the overlap of top100 significantly SAL-regulated genes between LNCaP and C4-2 cells. **F** Heat map represents the 33 genes upregulated upon SAL in both LNCaP and C4-2 cells. Color-key number represents normalized count. **G** Normalized log2 fold change of lncRNASAT1 upon SAL or AKTi + SAL in both LNCaP and C4-2 cells of RNA-seq data. **H** Confirmed regulation of the lncRNASAT1 using qRT-PCR in both LNCaP and C4-2 cells. **I**, **J** Concentration-dependent induction of the lncRNASAT1 by androgens using (**I**) R1881 or (**J**) DHT in LNCAP cells treated for 72 h by qRT-PCR. Values were normalized to TBP and GAPDH and relative expression was calculated compared with control treatment. Bars indicate the mean ± SEM (*n* = 4). *p ≤ 0.05, **p ≤ 0.01, ***p ≤ 0.001, ****p ≤ 0.0001, n.s. not significant.

**Fig. 6:**
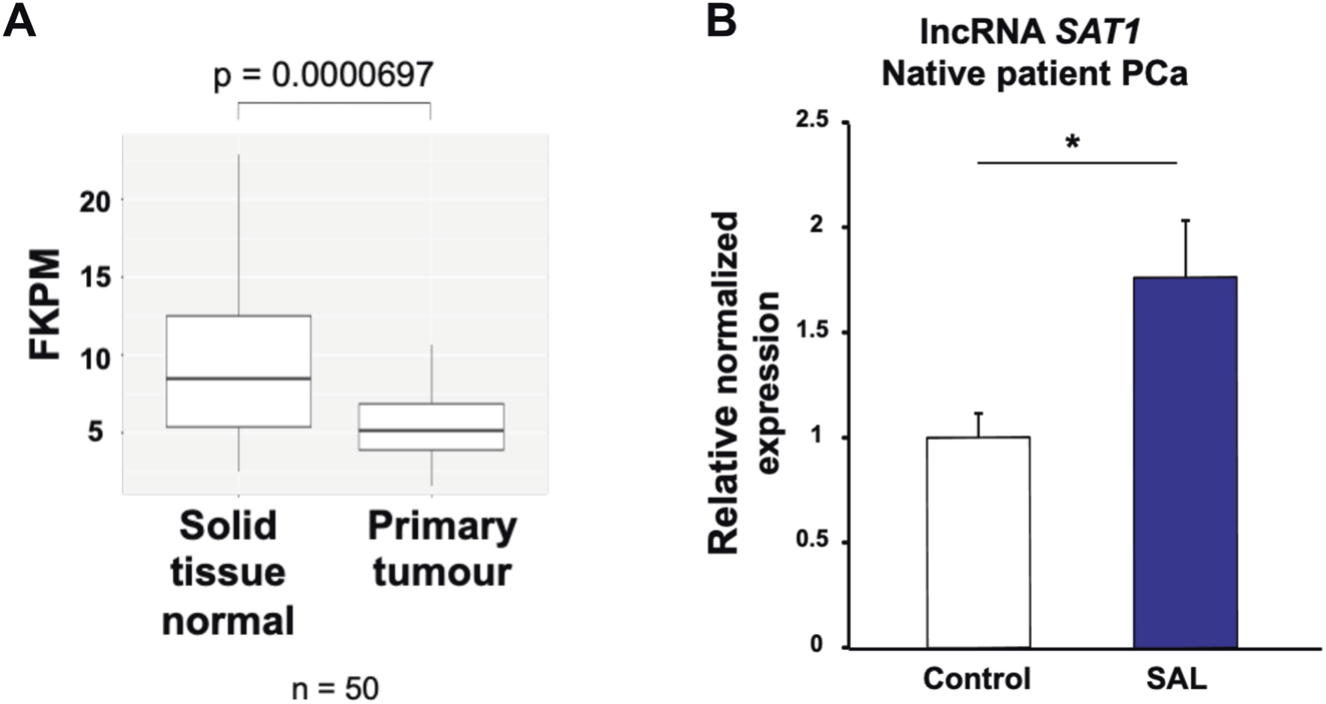
The expression of lncRNASAT1 is reduced in PCa and is inducible by SAL in tumor tissues. **A** TCGA database of 50 patient PCa samples, comparing the expression in nontumor and tumor areas, indicates that the expression of lncRNASAT1 is lower in primary tumor compared with normal tissues with a highly significant difference. **B** Human PCa tissues obtained from prostatectomy were treated with SAL ex vivo using 1 μM R1881. Analysis of the gene expression of lncRNASAT1 was performed by qRT-PCR. Values were normalized to the housekeeping gene TBP and indicated with the mean ± SEM (*n* = 7).

**Fig. 7:**
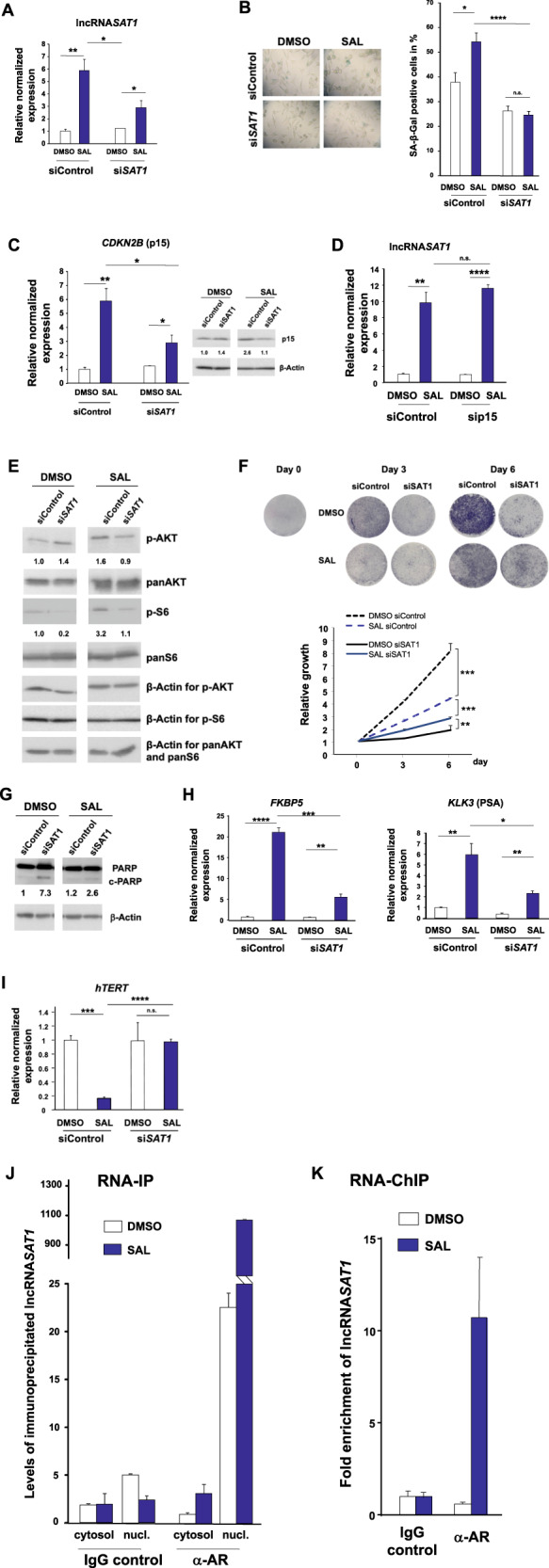
The lncRNASAT1 regulates CDKN2B/p15 and the Akt signaling to induce cellular senescence and control PCa growth. LNCaP cells were transfected with nontargeting siRNA (siControl) as negative control or siSAT1 targeting the additional exon in lncRNASAT1. After 24 h of transfection, cells were treated with SAL or 0.1% DMSO as solvent control for 48 h followed by RNA extraction. **A** The knockdown of lncRNASAT1 in LNCaP cells was confirmed with qRT-PCR (*n* = 3). **B** The level of cellular senescence was analyzed by quantification of SA-β-Galpositive stained LNCaP cells (*n* = 3). **C** The levels of CDKN2B mRNA and p15INK4B protein were analyzed with and without lncRNASAT1 knockdown (*n* = 3). **D** The level of lncRNASAT1 was analyzed in the CDKN2B-knockdown LNCaP cells using qRT-PCR (*n* = 3). **E** Changes of panAKT, p-AKT, panS6, and p-S6 levels after knockdown of lncRNASAT1 (*n* = 3). **F** Growth curves of LNCaP cells with and without SAL treatment comparing with and without knockdown of lncRNASAT1 analyzed by crystal violet staining (*n* = 2). **G** LncRNASAT1 suppression mediates apoptosis in LNCaP cells. Western blot analysis of cleaved PARP in response to lncRNASAT1 knockdown after 48 h of treatment with DMSO as solvent control or 1 nM R1881 (SAL). The protein expression was normalized to the loading control β-Actin using LabImage 1D software (*n* = 2). **H**, **I** qRT-PCR was used to analyze the expression of AR target genes through knockdown of lncRNASAT1 in LNCAP cells treated with and without SAL. Relative expression was calculated compared with solvent control. The mean ± SEM values were calculated from three independent experiments (*n* = 3). Indicated are (**H**) the positively androgen-regulated KLK3 and FKBP5 genes and (**I**) the negatively androgen-repressed hTERT gene. **J** LNCaP cells were incubated for 24 h with DMSO or SAL followed by cytosolic and nuclear extraction. Immunoprecipitation was performed using an AR antibody or a matched immunoglobulin G (IgG) as control. RNA-immunoprecipitated lncRNASAT1 was analyzed by qRT-PCR (*n* = 2). **K** RNA-ChIP was performed with cross-linked chromatin from LNCaP cells treated with DMSO or SAL for 24 h followed by immunoprecipitation of AR and qRT-PCR detection of the lncRNASAT1 (*n* = 4). *p ≤ 0.05, **p ≤ 0.01, ***p ≤ 0.001, ****p ≤ 0.0001, n.s. not significant.

**Fig. 8:**
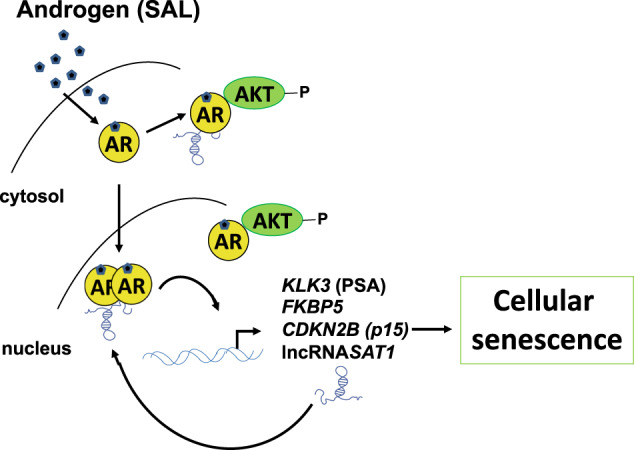
Schematic view of the AR-lncRNASAT1-AKT-p15 axis. SAL treatment induces the AR-AKT interaction and AKT phosphorylation, induction of p15, and cellular senescence. The knockdown of the lncRNASAT1 reduces AKT phosphorylation, the expression of the AR target genes encoding PSA and FKBP5, as well as p15INK4B protein level, leading to reduced level of cellular senescence. Further, the knockdown of p15INK4B reduces the SAL-induced cellular senescence.

The original article has been corrected.

