# Supplementary material for: The androgen receptor—lncRNASAT1-AKT-p15 axis mediates androgen-induced cellular senescence in prostate cancer cells
Source: Oncogene. 2021 Oct 19;41(7):943–59. doi: 10.1038/s41388-021-02060-5 (PMC8837536; doi:10.1038/s41388-021-02060-5)
Supplement: Supplementary file 1 — S1 [file 41388_2021_2060_MOESM1_ESM.pdf]

Supplemental Figure

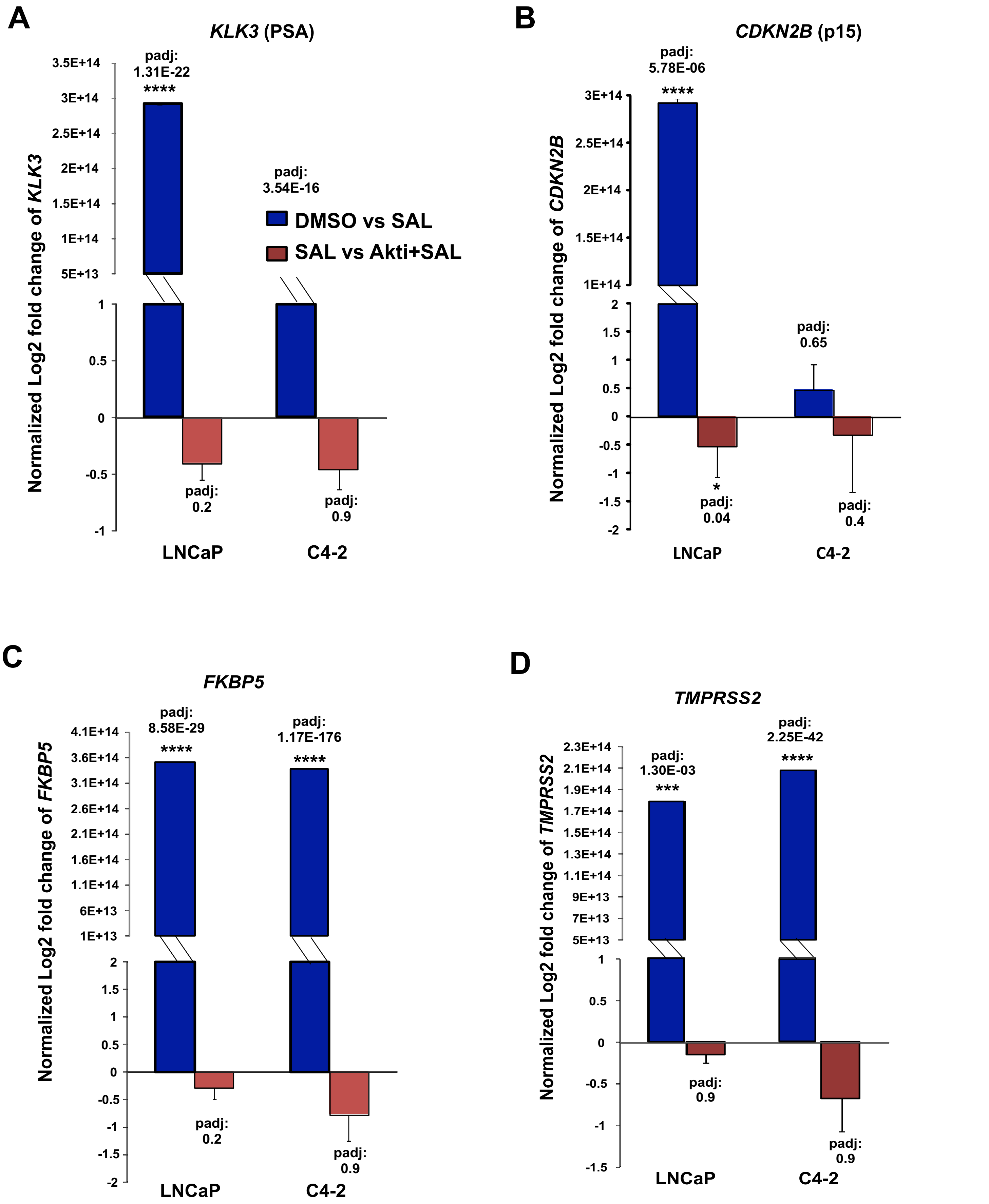

Fig. S1 Differential expression analysis indicates AKTi significantly alters expression of most genes regulated by SAL. Normalized log2 fold change and significance of expression by the treatments of (A) *CDKN2B* encoding p15, (B) *KLK3* encoding PSA, (C) *TEMPRSS2* and (D) *FKBP5* in both LNCaP and C4-2 cells. (n=3), ***padj≤0.001, ****padj≤0.0001
