# Supplementary material for: The androgen receptor—lncRNASAT1-AKT-p15 axis mediates androgen-induced cellular senescence in prostate cancer cells
Source: Oncogene. 2021 Oct 19;41(7):943–59. doi: 10.1038/s41388-021-02060-5 (PMC8837536; doi:10.1038/s41388-021-02060-5)
Supplement: Supplementary file 2 — S2 [file 41388_2021_2060_MOESM2_ESM.pdf]

Supplemental Figure

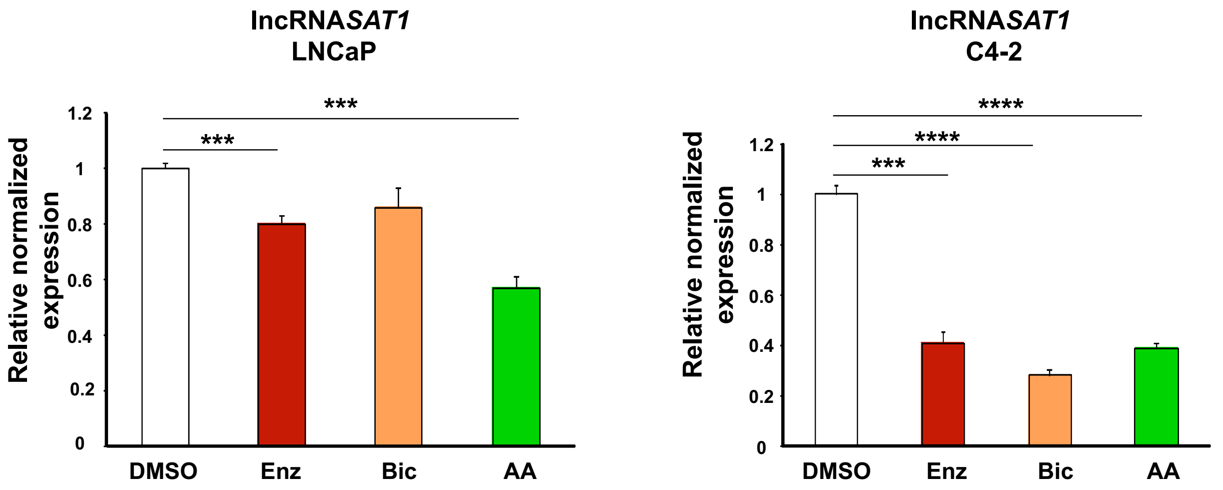

Fig. S2. The expression of lncRNA*SAT1* is repressed by AR antagonists*.* LNCaP and C4-2 cells were incubated for 72 h with DMSO as solvent control or different AR antagonist (Enzalutamide (Enz, 10 µM), Bicalutamide (Bic, 10 µM) and Atraric acide (AA, 100 µM). qRT-PCR was performed to analyze the expression of lncRNA*SAT1* in both LNCaP and C4-2 cells. Gene expression was normalized to *TBP* and *GAPDH3'* and the values for control samples. Error bars indicate the SEM of the mean of doublets. Three independent experiments were performed. Two-tailed unpaired Student's *t*-test was performed for statistical analysis (*p<0.05, **p<0.01, ***p<0.001) compared to DMSO treatment.
