# Supplementary material for: The androgen receptor—lncRNASAT1-AKT-p15 axis mediates androgen-induced cellular senescence in prostate cancer cells
Source: Oncogene. 2021 Oct 19;41(7):943–59. doi: 10.1038/s41388-021-02060-5 (PMC8837536; doi:10.1038/s41388-021-02060-5)
Supplement: Supplementary file 3 — S3 [file 41388_2021_2060_MOESM3_ESM.pdf]

Supplemental Figure

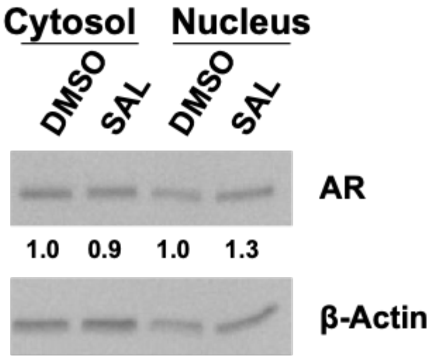

Fig. S3. Protein levels of AR in cytosol and nucleus. LNCaP cells were lysed after 24 h treatment with SAL and DMSO and separated into cytosolic and nuclear protein fractions. Western blot was performed to analyze AR levels. Values of control samples were set arbitrarily as 1. Quantification of bands was performed by LabImage D1 normalized to the loading control β-Actin.
