# Supplementary material for: The androgen receptor—lncRNASAT1-AKT-p15 axis mediates androgen-induced cellular senescence in prostate cancer cells
Source: Oncogene. 2021 Oct 19;41(7):943–59. doi: 10.1038/s41388-021-02060-5 (PMC8837536; doi:10.1038/s41388-021-02060-5)
Supplement: Supplementary file 4 — S4 [file 41388_2021_2060_MOESM4_ESM.pdf]

Supplemental Figure

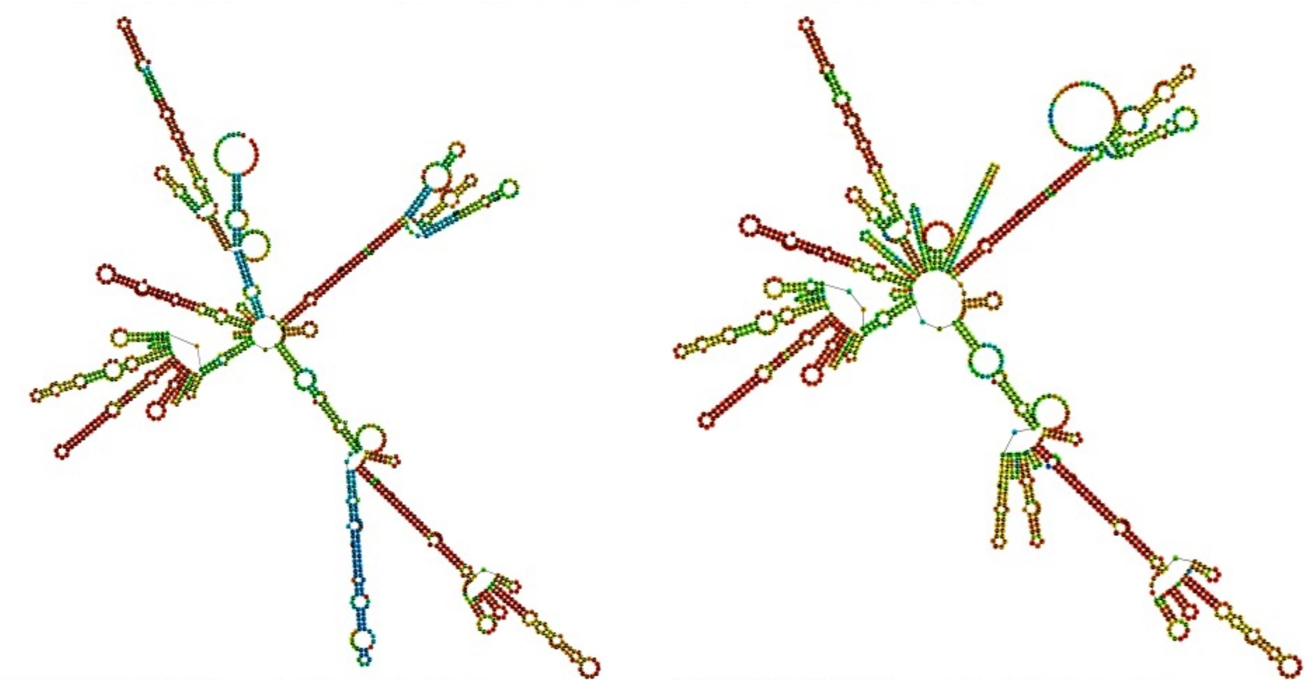

Fig. S4. Predicted structure of the lncRNA*SAT1*. Prediction of two likely secondary structures of lncRNA*SAT1* using the *RNAfold* online web server. The interaction probabilities between lncRNA*SAT1* and protein AR were calculated by http://pridb.gdcb.iastate.edu/RPISeq.
