# Supplementary material for: The androgen receptor—lncRNASAT1-AKT-p15 axis mediates androgen-induced cellular senescence in prostate cancer cells
Source: Oncogene. 2021 Oct 19;41(7):943–59. doi: 10.1038/s41388-021-02060-5 (PMC8837536; doi:10.1038/s41388-021-02060-5)
Supplement: Supplementary file 5 — Supplemental text [file 41388_2021_2060_MOESM5_ESM.docx]

**Supplement**

**Table 1: List of primary antibodies for Western blotting**

| **Target** | **Host** | **Dilution** | **Company** | **Cat. No.** |
| --- | --- | --- | --- | --- |
| AR | mouse | 1:2000 | BioGenex | AM256-5ME |
| β-Actin | mouse | 1:10000 | Abcam | Ab6276 |
| PARP | mouse | 1:2000 | Cell Signaling | 9546 |
| HSP-90 | rabbit | 1:1000 | Cell Signaling | 4877 |
| p15^INK4b^ | rabbit | 1:2000 | MyBioSource | MBS821044 |
| p-AKT (Ser473) | rabbit | 1:5000 | Cell Signaling | 4058 |
| pan-AKT | rabbit | 1:5000 | Cell Signaling | 4685 |
| p-S6 (S235/236) | rabbit | 1:5000 | Cell Signaling | 2211 |
| pan-S6 (5610) | rabbit | 1:5000 | Cell Signaling | 2217 |
|  |  |  |  |  |

**Immunofluorescence staining**

LNCaP cells were seeded in RPMI 1640 medium containing 5% FBS and cultured for 48 h. After 72 h of ligand treatment, cells were fixed with 4% paraformaldehyde and stained with 5 µg/ml wheat germ agglutinin (WGA) in HBSS for imaging of cellular membranes. Cells were permeabilized with 0.25% Triton-X100/PBS for 10 min at room temperature and after three washing steps in 1x PBS a blocking solution (5% Normal Goat Serum/PBS) was added for 1h. Primary antibodies were incubated in a humidified chamber overnight at 4°C. Rabbit anti-goat or goat anti-rabbit secondary antibodies were incubated for 1 h at room temperature. After washing, cells were stained with Hoechst in 1x PBS followed by mounting with Flouremount G. Images were obtained with a confocal laser scanning microscope (Zeiss LSM 880) with Airyscan in super resolution using a Plan-Apochromat 63x/1.4 oil DIC M27 objective confocal scanning fluorescence microscope. Fiji software was used for analysis of the images.

**Table 2: List of primary antibodies for immunofluorescence staining**

| **Antibody** | **Host** | **Dilution** | **Company** | **Cat. No.** |
| --- | --- | --- | --- | --- |
| AR | goat | 1:50 | Abcam | ab19066 |
| p-AKT (Ser473) | rabbit | 1:200 | Cell Signaling | 4058S |
| pan-AKT | rabbit | 1:100 | Cell Signaling | 4685S |

**RNA-sequencing and transcriptome analysis**

Total RNA was isolated from LNCaP and C4-2 cells treated for 72h with solvent control or androgens, in triplicate, using peqGOLD TriFast (Peqlab, Erlangen, Germany) according to the manufacturer’s protocol. For SAL 1 nM R1881 and 1 μM Akti were used as described earlier (Roediger et al., 2014). Sequencing library was prepared using SMARTer® Stranded Total RNA Sample Prep Kit - HI Mammalian (TAKARA, Kusatu, Japan). The paired-end sequencing was conducted using Illumina NextSeq 500/550 High-Output v2 Kit (150 cycles). Fastq files quality check was performed using FastQC (v0.11.5). The first 4 nucleotides were removed from the sequenced reads using fastx_trimmer (FASTX Toolkit 0.0.13). The low quality nucleotides from the end of each read were removed using fastq_quality_trimmer (FASTX Toolkit 0.0.13) with -Q 33 -t 20 -l 25 parameters. Only the first sequenced read from the pair end (R1) was used for the downstream analysis. The fastq files were mapped to the hg19 genome using tophat (v2.1.0) with the following parameters --bowtie1 --no-coverage-search -a 5. The number of reads covered by each gene was calculated by htseq-count (0.11.2) with -s no -a 0 -t exon -m intersection-nonempty parameters and hg19 gencode.v19 annotation. Before further analysis all the rRNA genes (5srRNA, rRNA, mt-rRNA) were removed from the count data. To calculate p-value and normalized count (based on the geometric library size factors), DESeq2 (1.20.0) R package with the default parameters and paired test was used. For gene ontology analysis, the deferentially expressed genes were used in gprofiler function from gProfileR (v0.7.0) R package, searching in the GO:BP, GO:MF, GO:CC and REAC data bases.

Gene set enrichment analysis (GSEA) of senescence was performed based on FRIDMAN_SENESCENCE_UP gene set containing 75 genes that are upregulated in senescent cells. Using the normalized counts and the custom gene set as input, the GSEA profile was plotted and q-value (False discovery rate, FDR) was calculated (GSEA, v4.1.0). According to the GSEA recommendation, the cutoff of 25% error for FDR is considered as significant (Mutha et al., 2003; Subramanian et al., 2005). Gene set enrichment analysis (GSEA) of senescence was performed based on FRIDMAN_SENESCENCE_UP gene set containing 75 genes which are upregulated in senescent cell. Using the normalized counts and the custom gene set as input, the GSEA profile was plotted and q-value (False discovery rate, FDR) was calculated (GSEA, v4.1.0). According to the GSEA recommendation, the cutoff of 25% error for FDR is considered as significant (2, 3).
